# Solvent and temperature effects in the photoiniferter RAFT polymerisation of PEG methacrylate†

**DOI:** 10.1039/d5py00300h

**Published:** 2025-05-28

**Authors:** Roujia Chang, Bryn D. Monnery, Inge S. Zuhorn

**Affiliations:** a Department of Biomaterials & Biomedical Technology, University Medical Center Groningen, University of Groningen Antonius Deusinglaan 1 9713 AV Groningen The Netherlands bryn.monnery@gmail.com i.zuhorn@umcg.nl

## Abstract

Photoiniferter (PI)-RAFT polymerization is a promising approach to synthesise a broad range of (meth)acrylic and styrenic polymers because of its highly ‘living’ nature. The lack of an imbalance between initiating and chain-transfer fragments minimises the inherent bimolecular termination of conventional RAFT. Poly(poly(ethylene glycol) methyl ether methacrylate) (P(PEGMA)) is a potential biocompatible material for biomedical applications, but the highly reactive free radical of PEGMA makes control of its polymerisation challenging. In this study, we investigated the synthesis of P(PEGMA) through PI-RAFT. Current studies on the PI-RAFT mechanisms are limited and the effect of solvents on kinetics has not been reported. We varied several reaction conditions: excitation wavelengths, monomer concentrations, temperatures, and solvents. The propagation constant (*k*_p_) values were affected by the RAFT main equilibrium. We calculated the Arrhenius parameters, enthalpy of activation (Δ*H*^‡^), and entropy of activation (Δ*S*^‡^) for polymerization in various solvents. Regression analysis was conducted to fit the results with extinction coefficients of CTA in seven common solvents, solvent physical properties, and solvatochromic scales. The effective collision factor *A* had a good fitting with an exponential regression model of the extinction coefficients, indicating a strong relationship between the reaction rate and excitation of the CTA. Solvent polarity scales, such as Kalmet–Abraham–Taft (KAT) and Catalan parameters, failed to predict *k*_p_, Arrhenius parameters, Δ*H*^‡^, and Δ*S*^‡^. A chain transfer constant *C*_tr_ > 1 for all syntheses indicated relatively good control over the polymerization through degenerative chain transfer with CTA radicals. In general, *C*_tr_ decreased with increasing temperatures, a result of the rate of excitation by photon absorption being constant, but the *k*_p_ being increased by the temperature. Anisole was the best solvent, able to keep *Đ* = 1.30 even at 40 °C.

## Abstract 2

Photoiniferter (PI)-RAFT polymerisation is a promising approach to synthesise a broad range of polymers due to its' "living" nature. We have investigated the effect of illumination, solvent and temperature on the PI-RAFT polymerisation of PEGMA.

## Graphical abstract

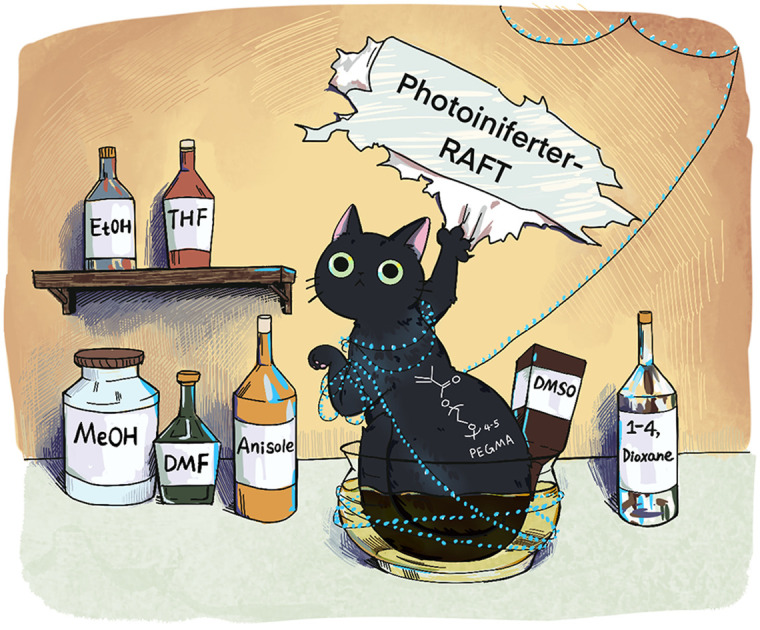

## Introduction

Poly(PEGMA) (P(PEGMA)) is a bottle-brush homopolymer that has gained great interest, especially in the field of biomedical application. Unlike hydrophilic polyethylene glycol (PEG), it is always in the brush conformation instead of the random coil conformation, enhancing stealth effects,^1–3^ which makes P(PEGMA) a potential candidate for fabricating hydrogels, micelles, nanogels, and nanoparticles for *in vivo* applications.^4–6^ Further, since the individual PEG chains are small, it may avoid the immunogenic effects of PEG, since a minimal number of repeat units are necessary to be recognised by anti-PEG antibodies. This minimum value has been variously reported as 4–5,^7^ 6-7,^8^ and 16 (when bound to a protein),^9^ but it appears the necessity is for an oxyethylene trimer to reach the binding site of the antibody.^10^ The only alternatives are to disrupt the PEG structure or to adopt an alternative polymer, such as poly(2-ethyl-2-oxazoline).^11^ In the case of PEGMA, PEGMA-300 (*M*_n_ of the PEG side chain *ca.* 200, or 4–5 EO units) is the shortest side-chain unit that is water soluble without lower critical solution temperature (LCST) effects, as the cloud point of Poly(PEGMA-300) is 55 °C.^12^ The bottlebrush side-chain is short enough and densely packed enough that reaching the antibody binding site is unlikely.

**Scheme 1:**
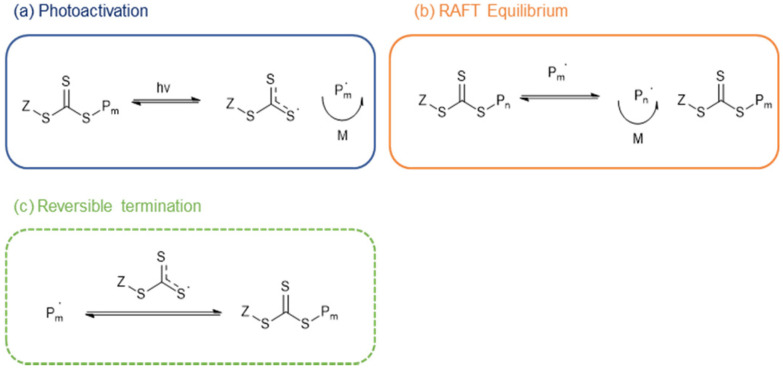
General mechanism of PI-RAFT polymerisation. The S–C bond is photolysed to generate a free radical (a, photoactivation). The propagating radical can either chain transfer the active centre (b, RAFT equilibrium) or combine with a photoiniferter radical to terminate reversibly (c, reversible termination).

Much attention has been given to reversable deactivation radical polymerisations,^13–15^ such as (1) atom transfer radical polymerisation;^16–18^ (2) nitroxide mediated polymerisation;^19–22^ and (3) reversable addition-deactivation chain transfer polymerisation.^19,23–27^ Conventional thermal RAFT typically shows pseudo-1^st^ order kinetics,^28^ but since it relies on an excess of R-groups over the CTA fragment to create propagating radicals, always shows some loss of livingness.^29^ To avoid this the radicals may be generated by a photocatalyst,^30–35^ or by the direct absorption of photons by the CTA to generate an excited state leading to photolysis and the formation of a propagating radical. This process is photoiniferter-RAFT (PI-RAFT, Scheme 1).

In PI-RAFT, which was first report by Otsu,^36,37^ the CTA is directly activated by either (ultraviolet light excited) n → π* or (near UV or visible light excited) π → π* transitions, leading to S–C bond homolysis.^38,39^ As expected of a RAFT process, it retains ‘living’ characteristics^40,41^ due to persistent radical effect,^38,42^ but is oxygen-tolerant.^43–45^ Trithiocarbonate, dithiocarbamate, and xanthate CTAs can be used as the photoiniferter, but^14,46^ trithiocarbonates are of particular interest as they are excited by blue light and polymerise rapidly. In contrast, whilst dithiocarbonates are amenable to blue-light polymerisation, it is very slow.^47^ Xanthates can be combined with other CTA's to achieve visible light PI-RAFT.^48^

The reaction kinetics of PI-RAFT are affected by the activation mechanism, wavelength, and intensity, with n → π* excitation being more efficient.^39^ With TTC's, green light still provides some activation, although it is less efficient than blue light.^49,50^ Without temperature control, high light intensity accelerates the rate of polymerisation,^41,50^ but a loses livingness.^51^

The application of photoiniferters has been extended to various monomers.^52^ However, there is no universal photoiniferter, and its selection matters to achieve good control in livingness and dispersity (*Đ*). Trithiocarbonates are common CTAs/photoiniferters for acrylates, acrylamides, and methacrylates and gives pseudo-first order kinetics for these monomers,^14,53–58^ but methacrylates give broader dispersities as they form stable free radicals with lower deactivation rate constants.^59–64^

In order to have a very narrow dispersity value similar to living anionic polymerisation, the number of propagation events per activation needs to be ≪1, and hence *C*_tr_ ≫ 1. A better understanding of the kinetics of PI-RAFT seems necessary.

**Fig. 1:**
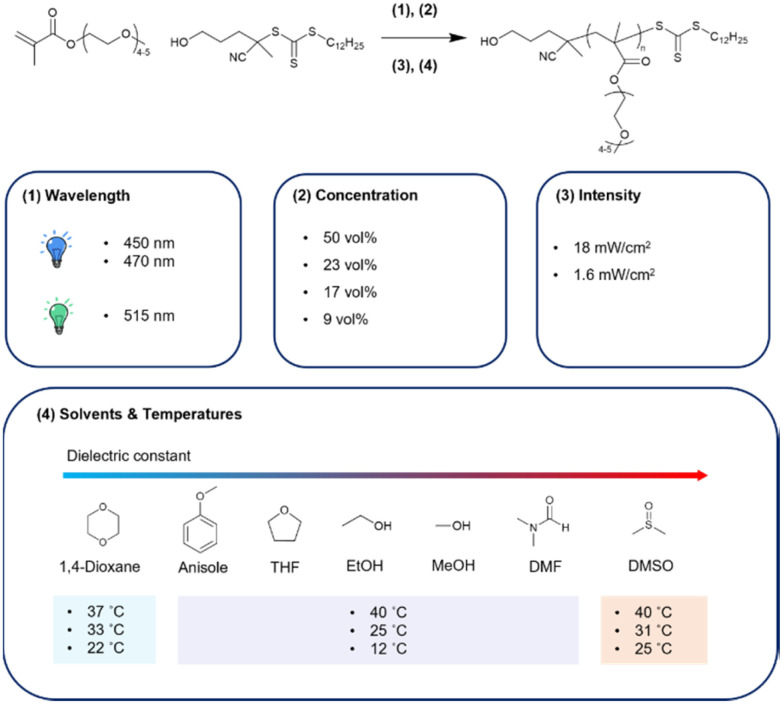
P(PEGMA) synthesis scheme through the PI-RAFT approach and overview of this study. The synthesis in 1,4-dioxane and DMSO cannot be conducted at 12 °C because of the high melting point of solvents.

In this work, which is aimed to investigate a better synthetic method for potential biomedical material P(PEGMA), we investigated the effect of excitation wavelength, intensity, temperature, concentration, and solvents on the PI-RAFT of PEGMA, *M*_n_ = 300 g mol^−1^ (Fig. 1).

## Experimental

### Determination of extinction coefficient

The absorbance of CTA between 300–500 nm in various solvents (DMSO, 1,4-dioxane, anisole, THF, EtOH, MeOH, and DMF) was measured by UV-Vis in a standard quartz cuvette. The extinction coefficients (*ε*) were determined through Beer–Lambert's law (eqn (1)).

1 *A* = *ε*·*c*·*l*

### Synthesis of P(PEGMA) under blue and green light

P(PEGMA) was polymerised under blue (*λ*_max_ = 470 nm, 1.6 mW cm^−2^) and/or green light (*λ*_max_ = 515 nm, 1.6 mW cm^−2^) irradiation. PEGMA was dissolved in DMSO (concentration of 50 vol%, 1.75 M) with [M]_0_ : [I] = 100, where [M]_0_ is the initial monomer concentration, and [I] is the concentration of photoiniferter. The solution was sparged with N_2_ for 1 h. The reaction was carried out at 22 °C under blue light for 1.5 h, green light for 4 h, and switching from blue light (0.5 h) to green light (2.5 h). The target conversion was 50%. The reaction mixture was dialyzed with a 3.5 kDa Spectra/Por 3 dialysis membrane against 1 L demineralized water for 2 d, with subsequent lyophilization to isolate the polymers. Kinetic data was collected at various time points and analysed with ^1^H NMR in CDCl_3_. The conversion was calculated from the methylene protons on the ethylene glycol chain for the remaining monomer (4.23 ppm) and the corresponding signal of polymers (4.01 ppm, eqn (2), indicated in Fig. S1†).2
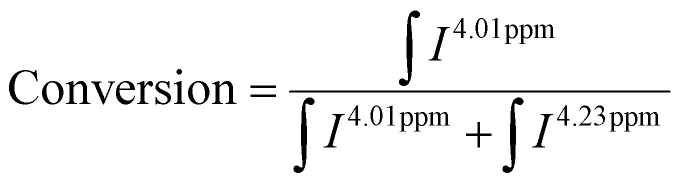


### Synthesis of P(PEGMA) at different concentrations

Three PEGMA concentrations in DMSO were used to investigate the effect of concentration on *Đ*: 23 vol% (0.81 M), 16.7 vol% (0.58 M), and 9 vol% (0.32 M). Reaction mixtures were prepared as above, varying the concentration. Polymerisations were performed at 22 °C under blue light irradiation (*λ*_max_ = 470 nm, 1.6 mW cm^−2^). The target conversion was 50%. The reaction mixture was dialyzed with a 3.5 kDa Spectra/Por 3 dialysis membrane against 1 L demineralized water for 2 d, with subsequent lyophilization to isolate the polymers. Kinetic data was collected at various time points and analyzed with ^1^H NMR in CDCl_3_.

### Synthesis of P(PEGMA) in different solvents and at three different temperatures

Seven solvents (1,4-dioxane, anisole, THF, EtOH, MeOH, DMF, and DMSO) were used to investigate the effect of solvent properties on PEGMA polymerisation. We performed synthesis at three temperatures: (1) 40, 32, and 22 °C in 1,4-dioxane; (2) 40, 25, and 12 °C in anisole, THF, EtOH, MeOH, and DMF; and (3) 40, 31, and 25 °C in DMSO. Because of an unavoidable fluctuation in the environmental temperature due to the lack of temperature control, the ability to control the reaction temperature was limited. The system was warmed by insulation and cooled by compressed air or using a cold room of 4 °C, whilst the temperature of the air surrounding the reactor was measured. PI-RAFT polymerisation of PEGMA in DMSO and 1,4-dioxane was conducted at a higher temperature (*ca.* 30 °C) due to the relatively high melting point of the solvents. PEGMA monomer was dissolved in solvents (concentration of 50 vol%, 1.75 M) with [M]_0_ : [I] = 100. The solution was degassed under N_2_ for 1 h. The reactions were performed under a blue light source (*λ*_max_ = 450 nm, 18 mW cm^−2^). For synthesis in anisole, THF, and 1,4 dioxane, the reaction mixture was precipitated in heptane (20× reaction volume) and further reprecipitated by dissolving in 1 mL DCM, followed by repeated precipitation in heptane (20× reaction volume). The isolated polymers were dried in a vacuum oven at room temperature. For synthesis in DMSO, DMF, EtOH, and MeOH, the reaction mixture (1–2 mL) was dialyzed with a 3.5 kDa Spectra/Por 3 dialysis membrane against 1 L demineralized water for 2 days, and subsequently lyophilised to isolate the polymers. Kinetic data was collected at various time points and analysed with ^1^H NMR in CDCl_3_.

### Determination of the propagation rate constant (*k*_p_)

Photoiniferters can be efficiently initiated upon irradiance at the wavelengths of interest, and the propagating radical concentration is assumed to be the concentration of photoiniferter ([I]), and the equilibrium between the active and inactive states is subsumed in *k*_p_. The values of *k*_p_ were determined by the reaction kinetics and eqn (3) (rearranged into eqn (4)),3
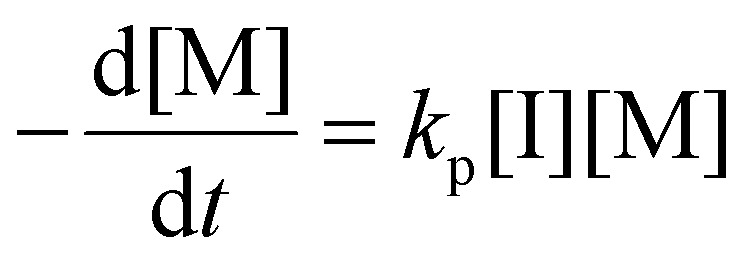
4
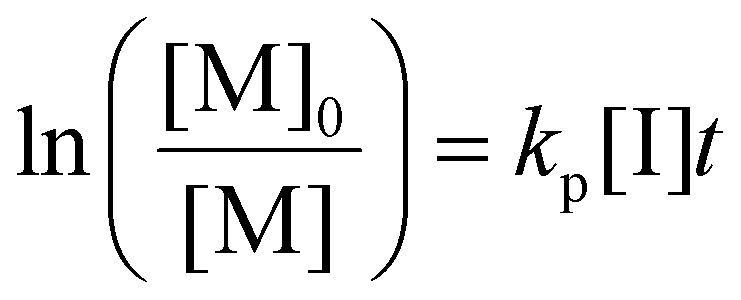
where 
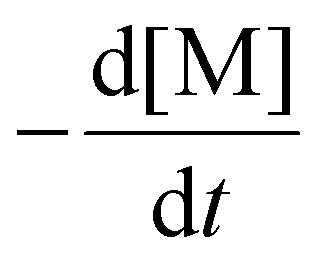
 is the rate of change of monomer concentration, [M] is the monomer concentration at an instant during the course of polymerisation, and [M]_0_ is the initial monomer concentration.

### Determination of chain transfer coefficient (*C*_tr_)

The *C*_tr_ value is defined as a ratio of the rate constant of deactivation by chain transfer toward a CTA (*k*_deact_) to the propagation rate constant (*k*_p_) (eqn (5)). Under the steady state assumption, *C*_tr_ can be estimated from the Muller's model (eqn (6)),^65–67^5
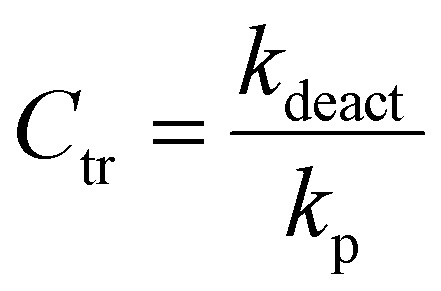
6
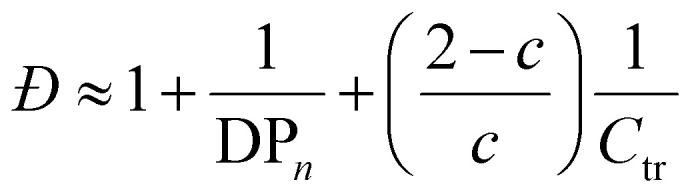
where DP_*n*_ is the number average degree of polymerization, and *c* is the monomer conversion.

### Determination of Arrhenius parameters

The *k*_p_ values were used to estimate the activation energy (*E*_a_) and the effective collision frequency (*A*) in various solvents using the Arrhenius equation (eqn (7) and its rearranged form eqn (8)). The *k*_p_ values at three temperatures were assumed to represent the expected linear correlation in eqn (8),7
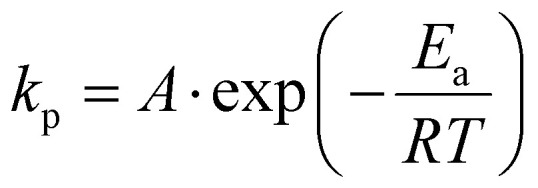
8
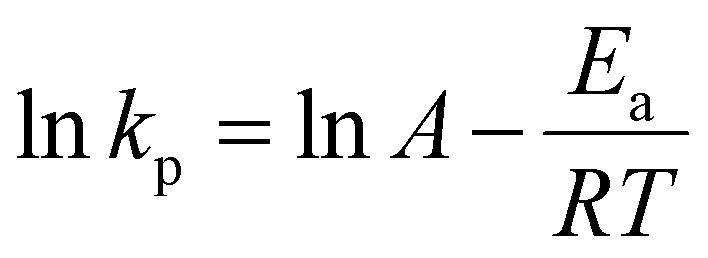
where *R* is the gas constant, and *T* is the temperature (in K).

### Determination of the enthalpy of activation (Δ*H*^‡^) and entropy of activation (Δ*S*^‡^)

The Δ*H*^‡^ and Δ*S*^‡^ values were calculated from the Eyring–Polanyi equation (eqn (9) and its rearranged form eqn (10)),9
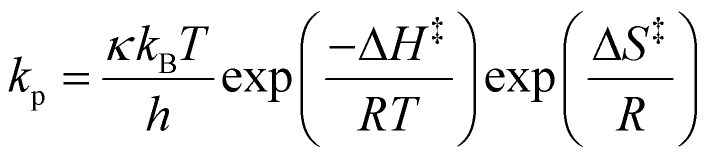
10
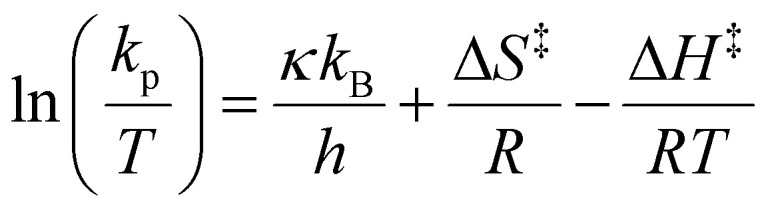
where *κ* is the transmission coefficient, k_B_ is the Boltzmann constant, and *h* is Planck's constant. *κ* was unity based on the assumption of irreversible propagation.^68^

### Simple linear regression with solvent properties

The values of *k*_p_, Arrhenius parameters, Δ*H*^‡^, and Δ*S*^‡^ were correlated with the extinction coefficient (*ε*) of the photoiniferter in various solvents, solvent physical properties (Table S1†), Hildebrand solubility parameters (*δ*_H_), and Dimroth *E*_T_(30) solvatochromic scale (Table S2†). Simple linear regression was carried out in Excel 2021.

### Multivariate linear regression of *k*_p_, Arrhenius parameters, Δ*H*^‡^, and Δ*S*^‡^ with Kalmet–Abraham–Taft (KAT) and Catalan solvatochromic scales

The values of *k*_p_, Arrhenius parameters, Δ*H*^‡^, and Δ*S*^‡^ were correlated with KAT scales (eqn (11)) and Catalan scales (eqn (12)). The parameters used in this study are outlined in Table S2.† Multivariate linear regression was carried out in Matlab 2020a.

11 *x* = *x*_0_ + *s*·π* + *a*·*α* + *b*·*β*

12 *x* = *x*_0_ + *p*·SP + *d*·SdP + *a*·SA + *b*·SB

## Results and discussion

### Verification of constant initiation

Before analysing rate constants *etc*., it must be verified that the proportion of CTA initiating chains is constant and ideally 100%. Plotting conversion against *M*_n_ measured by SEC (against PEG standards) shows extremely good correlation, and thus in all cases the initiation efficiency is the same (Fig. S2a†). It is extremely unlikely that such a correlation would result in any circumstances except ≈100% initiation efficiency. However, there was some induction, which was typically 5–10 min, but up to 20 min in one instance. This, we assume, was due to the last traces of oxygen being consumed by the radicals, but given the consistency of the molecular weight, we assume the loss of CTA in the induction phase is ≪1%. Plotting observed *M*_n_*vs.* PEG standards against theoretical *M*_n_ (Fig. S2b†) gives a strong relationship with a gradient of 0.7156. Since P(PEGMA) is a brush copolymer which is expected to be less expanded in DMF than PEG, this is also consistent with full initiation. Hence it is assumed that initiation efficiency is ≈100%.

### Effect of wavelength on PI-RAFT of PEGMA

**Fig. 2:**
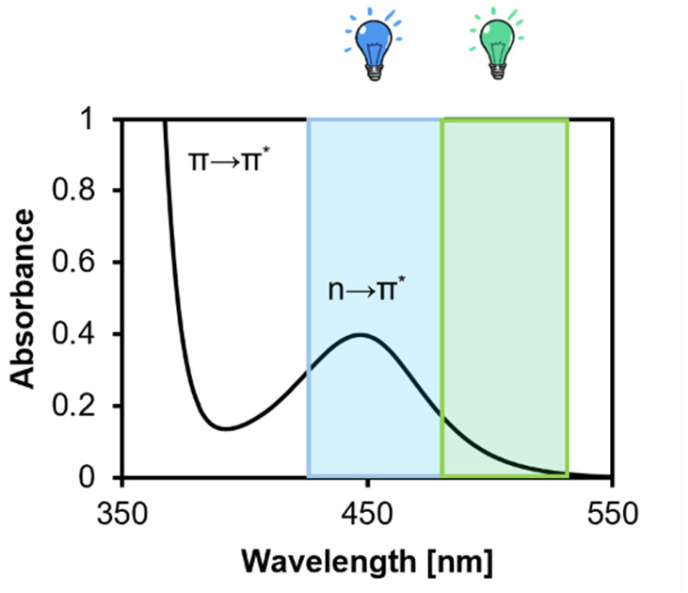
Absorbance of CTA in DMSO, 5 mg mL^−1^. π → π* and n → π* transition as annotated in the spectrum. Shaded areas indicate the overlap of the excitation mechanism with wavelengths of light used in this study.

**Fig. 3:**
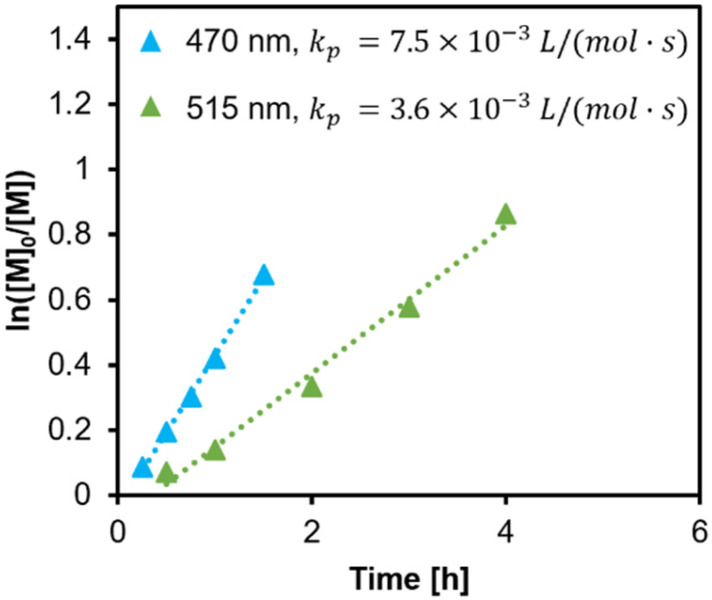
Polymerisation kinetics initiated under *λ*_max_ = 470 and 515 nm, 1.6 mW cm^−2^ in DMSO at a monomer concentration of 50 vol%, [M]_0_/[I] = 100, 22 °C.

Since, in general, slower polymerisations are more controllable, we aimed to decrease the rate of polymerisation by changing the wavelength of incident light from blue to green light (*λ*_max_ = 515 nm, 1.6 mW cm^−2^), which has a lower absorbance for the CTA (Fig. 2). At an ambient temperature of 22 °C, the rate of polymerisation was half that as under blue irradiation (*λ*_max_ = 470 nm, 1.6 mW cm^−2^) (Fig. 3). An increase in the induction period was observed, from negligible (*ca.* 5 min, which may be the temperature equilibrating) under blue light to *ca.* 21 min under green light.

We further switched light sources during the course of polymerisation, with blue light irradiance for 0.5 h and green light irradiance for 2.5 h to reach a targeted conversion of 50%. Nevertheless, *Đ* turned out to be similar (Table 1 and Fig. S4†).

**Table 1:** Synthesis of P(PEGMA) excited by different wavelengths (1.6 mW cm^−2^, 22 °C, 50 vol%)

No.	*λ*_max_ [nm]	Conv. [%]	*M*_n, theo_ [kDa]	*M*_w, SEC_ [kDa]	*M*_n, SEC_ [kDa]	*Đ*	*C*_tr_
1	470	49.2	14.8	26.6	20.7	1.28	11.60
2	515	57.9	17.4	32.6	24.3	1.34	7.47
3	470 → 515	40.0	11.7	21.8	16.3	1.34	12.05

In this study, we estimated *k*_p_ assuming fully initiated CTA through photolysis, and there were no observable signs of termination. A lower *k*_p_ value under green light can be explained by the involved equilibria (Scheme 1). Since photons carrying a lower energy are less efficient in exciting n → π* transition of the intermediates, the activation is slowed down.

### Effect of concentration on PI-RAFT of PEGMA

**Fig. 4:**
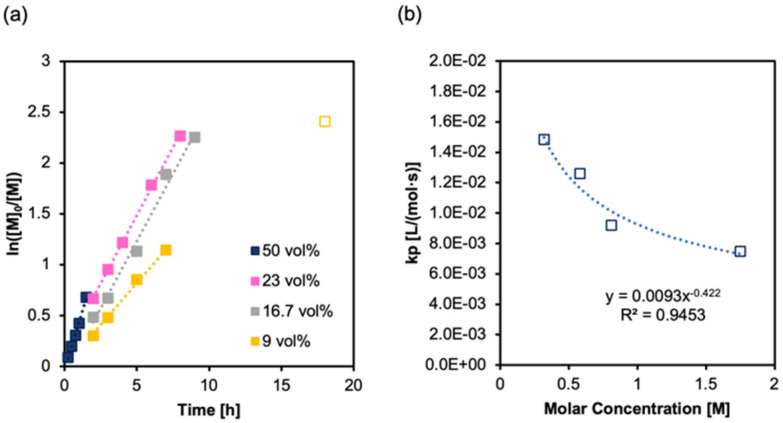
(a) Polymerisation kinetics at different monomer concentrations (50, 23, 17, and 9 vol%) in DMSO. Reaction kinetics at 50% v/v is polymer 1 reported in Fig. 3 and Table 1. Initiated under *λ*_max_ = 470 nm, 1.6 mW cm^−2^ in DMSO, [M]_0_/[I] = 100, 22 °C. The 18 h datapoint at 9% v/v is off trend and is excluded as an outlier, probably caused by the loss of active chain-ends. (b) Monomer concentration plotted against *k*_p_.

The most direct method to manipulate polymerisation kinetics is to vary the monomer concentration. We synthesised P(PEGMA) at three additional concentrations, ranging from 23 to 9 vol% (1.05–0.35 M) under blue light irradiance (*λ*_max_ = 470 nm, 1.6 mW cm^−2^) (Table 2). The RAFT process consists of four major processes of interest: (1) activation, (2) propagation, (3) deactivation, and (4) termination. Since propagation is proportional to the monomer concentration, the propagation (2) rate will be significantly slowed upon dilution. Termination (4) would typically be observed by tails and shoulders in the distribution (SEC), and a curvature in the kinetic plots. We have not observed these in the SEC (Fig. S5†), and only one datapoint in the kinetics suggested termination (likely a slow thiol degradation)^69^ and required exclusion, whilst all other points are linear and show good control (Fig. 4a). Whilst the rate decreases with lowered concentration, the *k*_p_ actually increases, and does so in a relationship close to *e* (Fig. 4b). This can be explained be the absorption of photons; as the concentration decreases, the decrease in light intensity through the beam path of the reactor is less, and so the average number of photons colliding with each CTA is higher.

**Table 2:** Synthesis of P(PEGMA) at different concentrations (*λ*_max_ = 470 nm, 1.6 mW cm^−2^, 22 °C)

No.	[M]_0_ [%v/v]	*k*_p_ [L (mol s)^−1^]	Conv. [%]	*M*_n, theo_ [kDa]	*M*_n_/*M*_w, SEC_ [kDa]	*Đ*	*C*_tr_
4	23	9.20 × 10^−3^	89.6	26.9	37.6/47.7	1.27	4.66
5	17	1.26 × 10^−2^	89.5	26.8	37.4/47.2	1.26	4.96
6	9	1.48 × 10^−2^	90.1	27.3	38.1/48.6	1.28	4.40

Activation/deactivation relates to the concentration of propagating radicals, which has significant effect on the kinetics, and affects termination. It will have very little effect on the dispersity since, for a given [M]_0_ : [I], the probability of a collision between a propagating end and either a monomer or the CTA fragment will be relatively the same. In fact, the molecular weight distribution is largely dependent on the ratio of propagation to deactivation events. As a consequence of each activation, the active radical will propagate a number of times before being deactivated by the CTA fragment. This will be a Poisson distribution with *R*_p_/*R*_deact_ being the Poisson parameter *λ* of the individual distribution. However, during the polymerisation process the activation-propagation-deactivation cycle occurs many times. Ergo, the Poisson distribution becomes compounded, and thus becomes a Gamma distribution.

### Effect of solvent on PI-RAFT of PEGMA

**Fig. 5:**
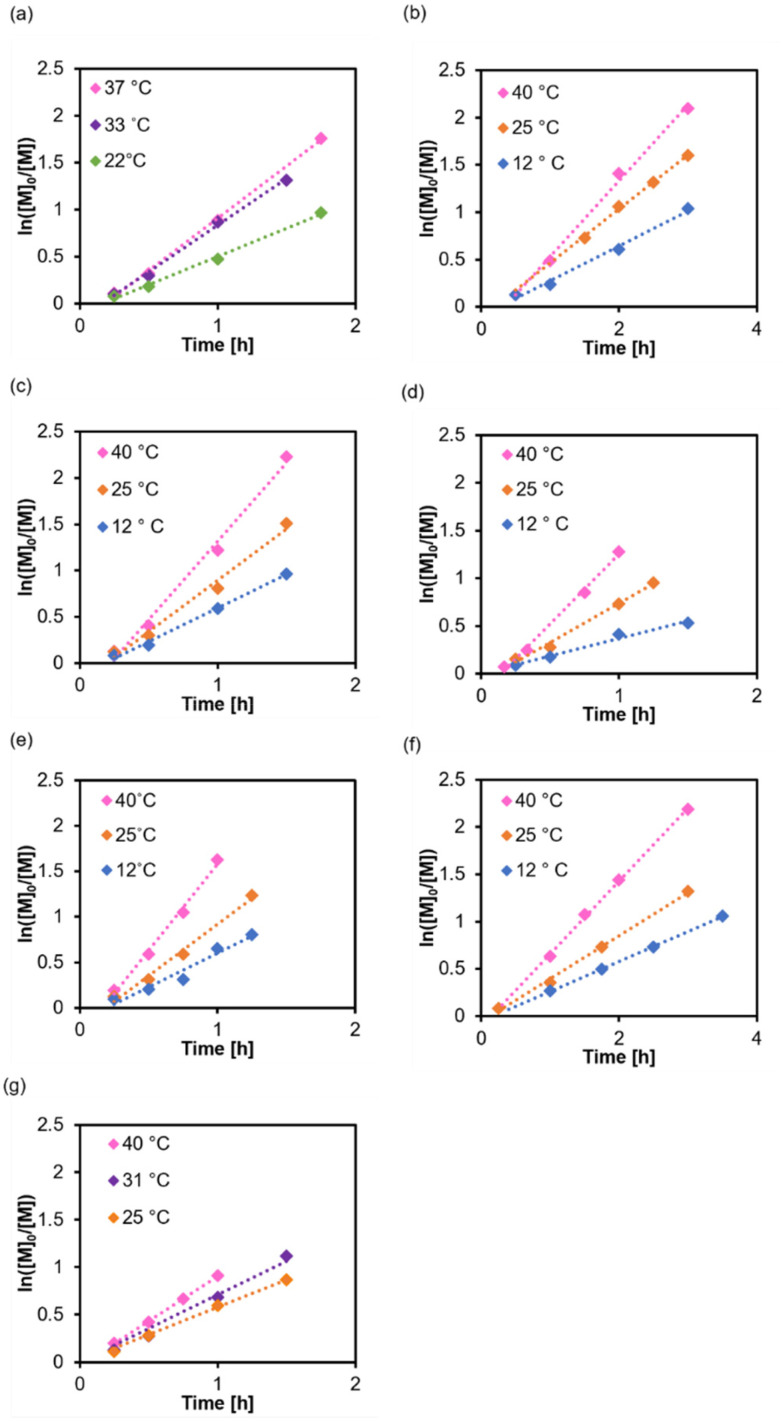
Polymerisation kinetics at different temperatures and in various solvents: (a) 1,4-dioxane, (b) anisole, (c) THF, (d) EtOH, (e) MeOH, (f) DMF, and (g) DMSO. Initiated under *λ*_max_ = 450 nm, 18 mW cm^−2^, [M]_0_/[I] = 100, [M]_0_ = 50 vol%.

To further understand how RAFT equilibria affect *k*_p_, we need to vary the Poisson parameter *λ* (*R*_p_/*R*_deact_) by changing the activation energy *E*_a_ (eqn (7) and (8)). One way of doing this is to alter the solvent. The n → π* transition of CTA in various solvents (Fig. S6†) had a similar full width at half maximum and peaked at 447–448 nm, where the extinction coefficients were determined (Fig. S7†). We synthesised P(PEGMA) at three temperatures (Fig. 5), and by inspection, *k*_p_ seems to be rather sensitive to the temperature. At 40 °C, the dispersity is wider for synthesis in most solvents because of the rapid propagation (Table 3, Fig. S8 and S9†). We used a blue light source higher in intensity (*λ*_max_ = 450 nm, 18 mW cm^−2^), and polymer 27 in DMSO had a faster rate of propagation compared to polymer 1. This has been reported in many studies,^38,50,70^ and it is intuitive that more absorbed photons generate more of the active species. Nevertheless, the comparison in this study is indirect due to different reaction temperatures.

**Table 3:** Synthesis of P(PEGMA) in various solvents and temperatures (*λ*_max_ = 450 nm, 18 mW cm^−2^, 50 vol%). Only final materials are shown

No.	Temp. [°C]	Reaction time [min]	Conv. [%]	*k*_p_ [L (mol s)^−1^]	*M*_n, theo_ [kDa]	*M*_w, SEC_ [kDa]	*M*_n, SEC_ [kDa]	*Đ*	*C*_tr_
**1,4-Dioxane**									
7	37	105	82.8	1.8 × 10^−2^	24.8	50.9	34.6	1.47	3.05
8	33	90	73.2	1.6 × 10^−2^	22	44.7	30.7	1.45	3.95
9	22	105	61.9	9.5 × 10^−3^	18.6	33.1	26	1.27	8.61
**Anisole**									
10	40	180	87.8	1.3 × 10^−2^	26.3	49.2	36.8	1.34	3.84
11	25	180	73.1	9.1 × 10^−3^	23.9	43.2	33.3	1.3	5.15
12	12	180	64.5	5.9 × 10^−3^	19.4	35	27.1	1.29	7.45
**THF**									
13	40	90	89.2	2.7 × 10^−2^	26.8	57.7	37.4	1.54	2.34
14	25	90	77.8	1.8 × 10^−2^	23.4	48.5	32.7	1.49	3.25
15	12	90	61.9	1.2 × 10^−2^	18.6	35.9	26	1.38	6.04
**EtOH**									
16	40	60	72.1	2.3 × 10^−2^	21.6	43.0	30.2	1.42	4.34
17	25	75	61.4	1.3 × 10^−2^	18.4	35.0	25.7	1.36	6.54
18	12	90	47.2	5.8 × 10^−3^	14.2	26.8	19.8	1.35	9.72
**MeOH**									
19	40	60	80.3	3.2 × 10^−2^	24.1	50.7	33.7	1.51	2.99
20	25	75	71	1.8 × 10^−2^	21.3	43.9	29.8	1.48	3.87
21	12	75	55.4	1.2 × 10^−2^	16.6	33.1	23.2	1.43	6.32
**DMF**									
22	40	180	88.8	1.2 × 10^−2^	26.6	51.8	37.2	1.4	3.18
23	25	180	73.2	7.2 × 10^−3^	22	40.4	30.7	1.32	5.61
24	12	210	65.2	5.0 × 10^−3^	19.6	34.8	27.4	1.27	8.02
**DMSO**									
25^a^	40	60	63.6	1.51 × 10^−2^	19.1	37.7	26.7	1.41	5.37
26	31	90	67.3	1.27 × 10^−2^	20.4	36.3	27.6	1.31	6.48
27	25	90	58	9.8 × 10^−3^	17.4	31.3	24.3	1.29	8.82

a The kinetics and SEC for no. 25 are from two equivalent experiments carried out under identical conditions.

**Fig. 6:**
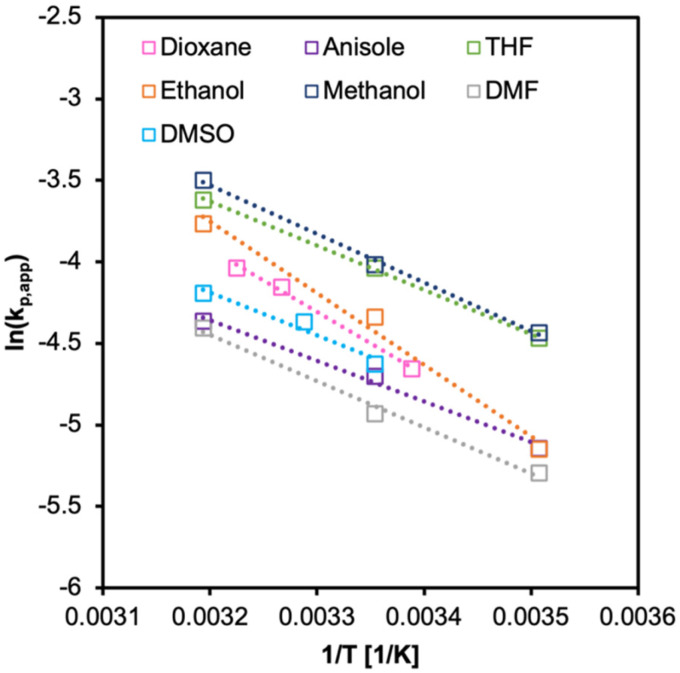
Arrhenius plot for PI-RAFT synthesis of PPEGMA in various solvents. Initiated under *λ*_max_ = 450 nm, 18 mW cm^−2^, [M]_0_/[I] = 100, 50 vol%.

**Fig. 7:**
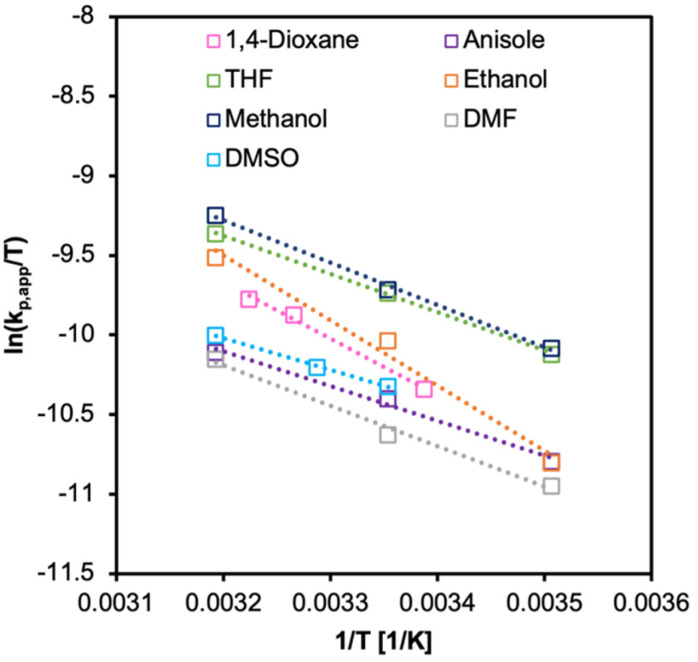
Eyring–Polanyi plot for PI-RAFT synthesis of PPEGMA in various solvents. Initiated under *λ*_max_ = 450 nm, 18 mW cm^−2^, [M]_0_/[I] = 100, 50 vol%.

From Arrhenius relationship eqn (7) and (8), *E*_a_ and *A* were calculated. The Δ*H*^‡^ and Δ*S*^‡^ values were calculated from Eyring–Polanyi equation (eqn (9) and (10)). The results are summarized in Fig. 6, 7, and Table 4. The values of *E*_a_ estimated from *k*_p_ were similar in all solvents but still surprisingly low. Notably, the effective collision frequency *A* for EtOH and 1,4-dioxane was orders higher in magnitude compared to the other solvents. The highly negative values of Δ*S*^‡^ suggest an associative pathway involved in the formation of the activation complex.

**Table 4:** Arrhenius parameters, enthalpy of activation (Δ*H*^‡^), and entropy of activation (Δ*S*^‡^) for PI-RAFT synthesis of PPEGMA in various solvents (*λ*_max_ = 450 nm, 18 mW cm^−2^, 50 vol%)

Solvent	*E*_a_ [kJ mol^−1^]	*A* [L (mol s)^−1^]	Δ*H*^‡^ [kJ mol^−1^]	Δ*S*^‡^ [J (mol K)^−1^]
1,4-Dioxane	31.95	4.33 × 10^3^	29.49	−183.54
Anisole	20.64	36.05	18.16	−223.44
THF	22.53	154.56	20.04	−211.36
EtOH	36.56	3.03 × 10^4^	34.07	−167.46
MeOH	24.73	397.86	22.25	−203.47
DMF	23.64	104.99	21.16	−214.55
DMSO	21.98	71.72	19.44	−217.9

To deeper understand the reaction mechanism, we did simple linear regression to fit the data with physical properties, *e.g.*, extinction coefficient *ε*, viscosity *η*, dipolarity, and dielectric constant (Table S3†). Very little correlation was observed. Dimroth *E*_T_(30) polarity scale in combination with the KAT equation is a useful approach to linear solvation energy relationship (LSER) analysis. Czerwinski was the first to apply LSER analysis to investigate the solvent effect on the free radical, where ln(*k*_p_) was a function of *E*_T_(30).^71^ LSER predictions were very successful for free radical polymerisation in highly polar solvents,^72,73^ but *E*_T_(30) scale is limited to describing dipolarity and hydrogen bond acidity of solvents. Hildebrand solubility parameter *δ*_H_ derived from cohesive energy of cavity formation also failed to fit to our results. The *δ*_H_ parameter was later introduced into the corrected KAT equations, but it overlaps with the dipolarity/polarizability term π* in eqn (12),^74^ which we will discuss later.

**Fig. 8:**
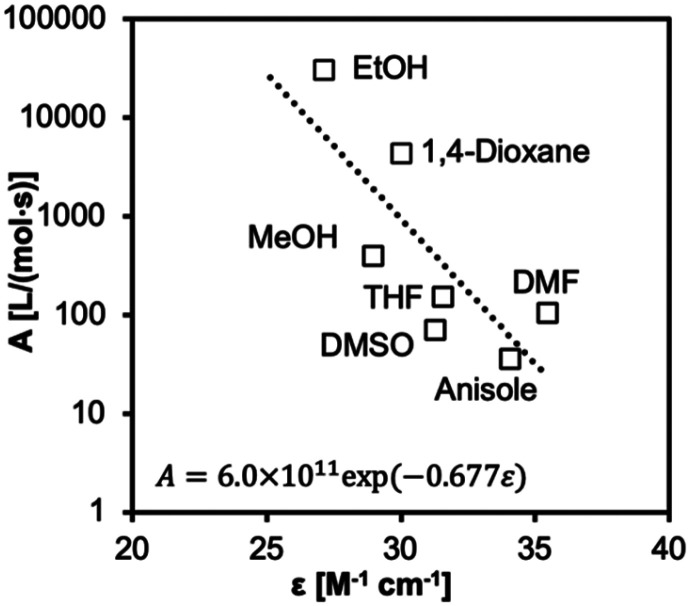
Pre-exponential factor *A* fitted to an exponential regression model correlated to the extinction coefficients *ε* (*r*^2^ = 0.93 to 2 d.p.).

A weak correlation with extinction coefficient *ε* was found for *k*_p_ and *E*_a_, but a moderate correlation to Δ*H*^‡^ and Δ*S*^‡^. The pre-exponential factor *A* was fitted exponentially to the *ε* values with an adjusted *R*^2^ of 0.91 (Fig. 8). A small decrease in the photons absorbed led to an exponential increase in the effective collisions of forming an activation complex. Fewer photons are absorbed to efficiently excite the n → π* transition. Therefore, more photons are available to photolyse intermediates into propagating free radicals. The increased number of collision events result from an increased concentration of active radicals.

The *k*_p_ values had a moderate correlation with the refractive index *n*_D_. When incident light passes through the interfaces, the solvent with a larger refractive index will cause a stronger scattering event and, therefore, a lower *k*_p_.

**Fig. 9:**
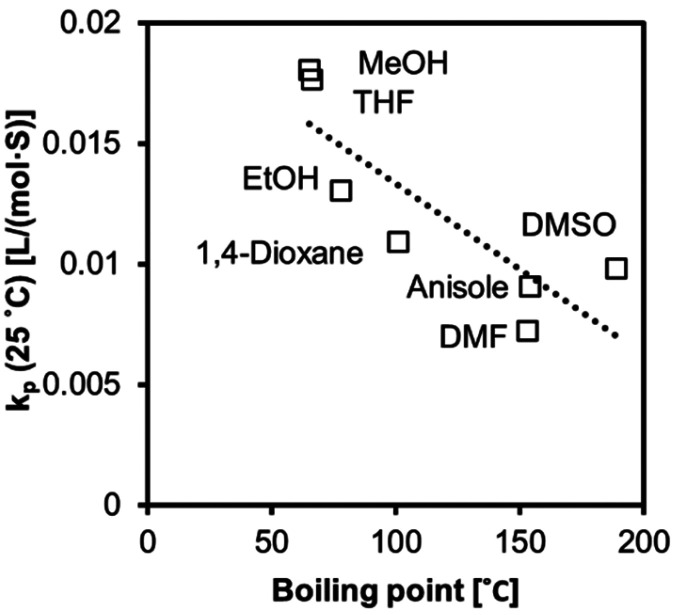
The values of *k*_p_ at 25 °C (*r*^2^ = 0.71) fitted to a linear regression model correlated to boiling points.

**Fig. 10:**
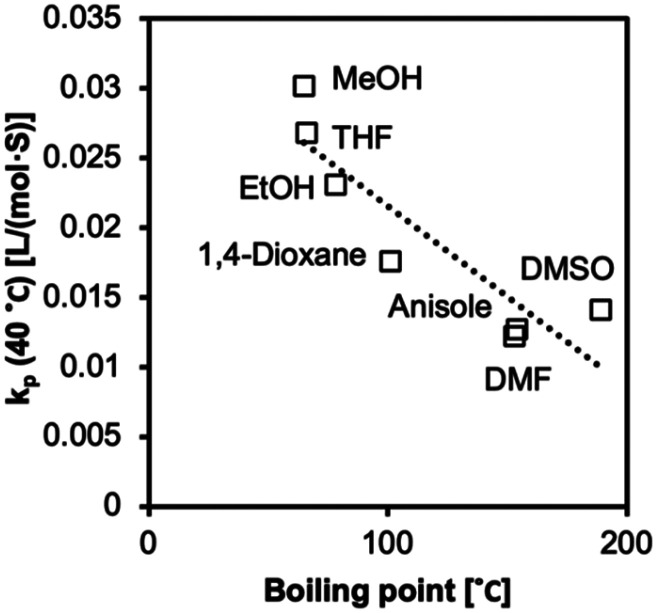
The values of *k*_p_ at 40 °C (*r*^2^ = 0.77) fitted to a linear regression model correlated to boiling points.

We observed increased *k*_p_ values with a decrease in boiling point (Fig. 9 and 10). The molecules in low-boiling point solvents are more kinetically energized, and radicals can easily escape from the solvent cage. The less successful prediction for *k*_p_ and *E*_a_ is likely due to its complication with the equilibria, as we discussed earlier in the text. Hoogenboom *et al.* reported an unexpectedly high reactivity of PEGMA monomer (*M*_n_ = 1100 g mol^−1^) that also indicated a hybrid *k*_p_, with an apparent chain transfer constant *C*_tr_ < 1.^75^

The KAT (eqn (11)) and Catalan (eqn (12)) solvatochromic scales are useful tools to study the effect of solvent on reaction kinetics. The KAT equation was constructed with acidity (*α*), basicity (*β*), and a conflated term of dipolarity/polarizability (π*). The term *x* is observed properties, *e.g.*, activation energy, and the natural logarithm of equilibrium constant. Multivariate linear regression using the KAT equation was statistically insignificant (Table S4 and Fig. S10†). Since the KAT scale is limited to specific interactions, namely acid–base interactions involving hydrogen bonding, Catalán proposed another solvatochromic scale that stresses nonspecific interactions. In the Catalan scale, except for acidity (SA) and basicity (SB), contributions from nonspecific interactions were isolated into dipolarity (SdP) and polarizability (SP) terms. Monnery *et al.* first applied the Catalan scale to predict the physiochemical properties of cationic ring opening polymerisation of 2-ethyl-2-oxazoline.^76^ Luo *et al.* extended the Catalan scale to the living anionic polymerisation of styrene.^77^ However, regression results turned out to be less significant (Table S5 and Fig. S11†).

**Fig. 11:**
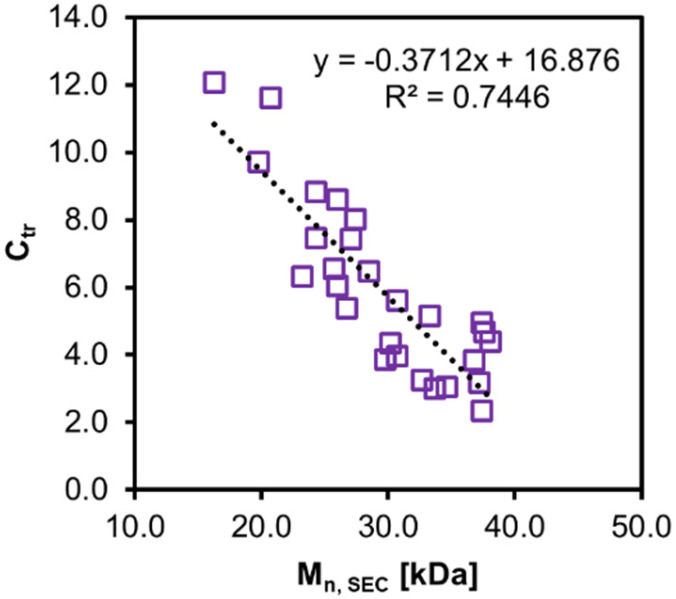
*C*_tr_ *vs. M*_n, SEC_ for P(PEGMA) 1–27.

The chain transfer constant *C*_tr_ (eqn (5)) was estimated from *Đ* using eqn (6). *C*_tr_ > 1 was observed for all the polymers 1–27, implying that deactivation is preferred compared to propagation, and thus PI-RAFT gains more control in P(PEGMA) synthesis compared to the conventional thermal RAFT, in which *C*_tr_ < 1.^75^ The *C*_tr_ values showed a negative correlation with *M*_n, SEC_ (Fig. 11). This can be attributed to diffusion-controlled deactivation.^78^ Benicewicz *et al.* determined decreasing diffusion coefficient of PMMA at higher molecular weights through diffusion-ordered spectroscopy (DOSY) ^1^H NMR.^79^ The *C*_tr_ values are generally lower at higher temperatures due to increasing *k*_p_ but are not significantly affected by solvents that influence the intrinsic *k*_p_ (Fig. S12†). The observed variation of *k*_p_ in different solvents is thus a result of changing activation rates.

### Mechanism

**Fig. 12:**
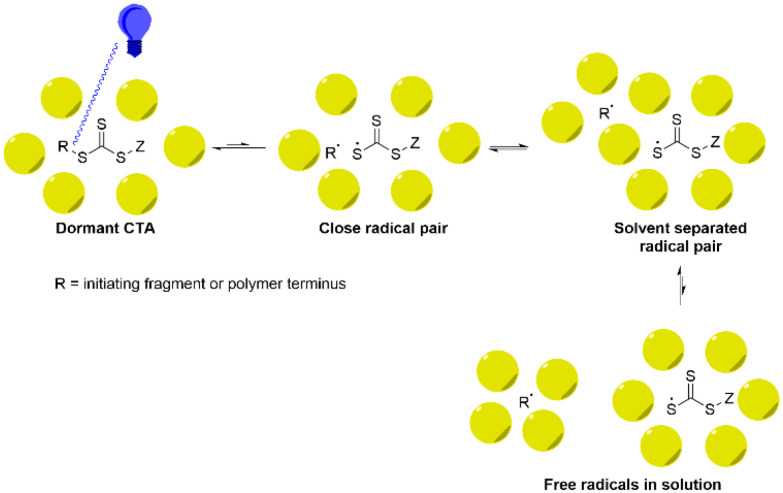
Cartoon showing suggested mechanism of activation and reactivation. The remodelling of the solvent shell to generate solvent separated radicals is necessary for propagation. However, moving too far to the right in this model creates a loss of fidelity.

In summary, this study indicates that the mechanism of PI-RAFT is not only dependent on the quantum yield of the excitation, which can be expected, but also on the kinetics of the separation of the radical pair generated by homolytic cleavage (Fig. 12). The radical pair initially generated will, unless the radicals become individually solvated, simply collapse back into the dormant pair. However, if they become solvent separated, then they may collide with monomers until they finally collide with another CTA fragment. The ease of this remodelling is reflected in the boiling point of the solvent and, if the remodelling allows for the easy escape, then the propagation rate will increase, the deactivation rate decreases and control of the polymerisation would be lost.

## Conclusion

For the synthesis of P(PEGMA) by PI-RAFT, we conducted a detailed study on various factors affecting the kinetics: wavelength, concentration, temperature, and solvents. For all the attempts, chain transfer constant *C*_tr_ > 1, indicating that a good control through degenerative chain transfer was maintained. The *k*_p_ values were affected by equilibria in each case, which explains the variation in the rate of polymerisation and molecular weight distribution. The CTA had a low absorbance in the green light, and the prolonged induction period and rate retardation indicated a lower quantum yield of excitation. We slowed down the rate of polymerisation by diluting the system. The molecular weight distribution that is closely linked to the kinetic chain length and was broadened by compounding of individual distributions. We further estimated the Arrhenius parameters, Δ*H*^‡^, and Δ*S*^‡^ in seven common solvents reported for P(PEGMA) synthesis. The *E*_a_ values were surprisingly low and accounted for the *k*_p_ values being sensitive to ambient temperature. Most importantly, the decreasing extinction coefficient of the CTA, that is solvent-dependent, exponentially increases the effective collision factor *A*. This might be attributed to more frequent activation. We further attempted to fit these values into KAT and Catalan equations. However, no relationship was revealed by the regression analysis, meaning these factors were not dominant. However, there was a good relationship with the boiling point, indicating the dominant feature was the remodelling of the solvation shell. This has not been considered before, and warrants further investigation.

## Author contributions

This paper was conceptualised by ISZ and BDM, with BDM developing the methodology. Investigation, formal analysis and primary writing were by RC, with BDM and ISZ supervising, reviewing and editing. Funding acquisition, data curation and administration were by ISZ.

## Conflicts of interest

There are no conflicts to declare.

## Supplementary Material

PY-016-D5PY00300H-s001

## Data Availability

Data for this article, including raw ^1^H NMR and SEC data are available at Zenodo, https://doi.org/10.5281/zenodo.15079075.
