# Supplementary material for: Solvent and temperature effects in the photoiniferter RAFT polymerisation of PEG methacrylate
Source: Polym Chem. 2025 May 28;16(25):2952–61. doi: 10.1039/d5py00300h (PMC12124182; doi:10.1039/d5py00300h)
Supplement: PY-016-D5PY00300H-s001 [file PY-016-D5PY00300H-s001.pdf]

Supplementary Information (SI) for Polymer Chemistry.
This journal is © The Royal Society of Chemistry 2025

Supporting Information

Solvent and Temperature Effects in the Photoiniferter RAFT Polymerisation of PEG Methacrylate

Roujia Chang,[1] Bryn D. Monnery,[1] Inge S. Zuhorn[1*]*

[1] Department of Biomaterials & Biomedical Technology, University Medical Center Groningen, 9713 AV, Groningen, the Netherlands.

Page	Content
**S1**	Table of Content
**S2**	Materials
**S2**	Instrumentation
**S3**	**Figure S1.** ^{1}H NMR spectra of PEGMA monomer and P(PEGMA) in DMSO solution, measured in $CDCl_3$.
**S3**	**Figure S2.**(a) Conversion plotted against $M_{n,\ SEC}$ calibrated against PEG standards for all SEC measurements in this manuscript. (b) Raw $M_{n,\ SEC}$ (vs PEG standards) results vs $M_{n,\ theoretical}$ plot.
**S3**	**Table S1.** Solvent physical properties.
**S4**	**Table S2.** Solvatochromic scales and Hildebrand solubility parameters
**S5**	**Figure S3.** ^{1}H NMR of P(PEGMA) in $CDCl_3$.
**S5**	**Figure S4.** (a) Molecular weight distribution of P(PEGMA) synthesised under different wavelengths. (b) Đ vs $M_{n,\ SEC}$ plot.
**S6**	**Figure S5.** (a) Molecular weight distribution of P(PEGMA) synthesised at different monomer concentrations. (b) Đ vs $M_{n,\ SEC}$ plot.
**S6**	**Figure S6.** Absorbance of CTA corresponding to the $n\rightarrow\pi^*$ transition in different solvents.
**S7**	**Figure S7.** Beer-Lambert plots for the $n\rightarrow\pi^*$ transition of CTA.
**S8**	**Figure S8.** Molecular weight distribution of P(PEGMA) synthesised at different temperatures and in various solvents.
**S9**	**Figure S9.** Đ vs $M_{n,\ SEC}$ plot of P(PEGMA) synthesised at different temperatures and in various solvents.
**S10**	**Table S3.** Adjusted R^2 values of simple linear regression.
**S10**	**Table S4.** Result of multivariate linear regression using the Kamlet-Abraham-Taft equation.
**S11**	**Figure S10.** Predicted values of k_p, Arrhenius parameters, enthalpy and entropy of activation from multivariate linear regression using the Kamlet-Abraham-Taft equation.
**S12**	**Table S5.** Result of multivariate linear regression using the Catalan equation.
**S13**	**Figure S11.** Predicted values of Arrhenius parameters, and enthalpy and entropy of activation from multivariate linear regression using the Catalan equation.
**S13**	**Figure S12.** C_{tr} vs $M_{n,\ SEC}$ for P(PEGMA) **7-27** synthesised at various temperatures and in 7 solvents.

Materials

Poly(ethylene glycol) methyl ether methacrylate (M_n = 300 g/mol, 300 ppm BHT and 100 ppm MEHQ as inhibitors), anisole (anhydrous, ≥99.7%), ethanol (EtOH, ≥99.5%, ACS reagent), and 4-cyano-4-[(dodecylsulfanylthiocarbonyl)sulfanyl]pentanol (CDP, ≥96.5%) were purchased from Sigma-Aldrich (Zwijndrecht, Belgium). 1,4-Dioxane (≥99.5%) was purchased from Honeywell (Fisher). Tetrahydrofuran (THF, ≥99.5%), n-heptane (≥99%), dichloromethane (DCM, ≥99%), aluminum oxide (Al_2O_3, ≥99%), and 3.5 kDa Spectra/Por 3 dialysis membranes were purchased from ThermoFisher (Bleiswijk, NL). Dimethyl sulfoxide (DMSO, ≥99%) was purchased from TCI (Zwijndrecht, Belgium). N,N-dimethylformamide (DMF, ≥99.8 %), deuterated chloroform ($CDCl_3$, ≥99.8 atom % D), and methanol (MeOH, ≥99%) were purchased from Carl Roth (Karlsruhe, Germany). LED strips were purchased from Okaphone (Groningen, NL).

Purification of Solvents and Monomer. PEGMA, 1,4-dioxane, and THF were purified through an alumina column to remove the stabilizers. DMSO, anisole, ethanol, methanol, and DMF were used as received.

Instrumentation

Proton Nuclear Magnetic Resonance (^{1}H NMR). ^{1}H NMR spectra were measured with a Bruker Avance 500 MHz NMR spectrometer. A number of 16 scans was collected. The chemical shifts are referenced to the residual undeuterated NMR solvent signal at 7.26 ppm ($CHCl_3$).

Size Exclusion Chromatography (SEC). Elution profiles of the polymers were obtained on a Waters e2695 Separations Module equipped with an Agilent PLgel 5 μm MIXED-D 300 × 7.5 mm column and Waters photodiode array detector (PDA 2998), fluorescence detector (FLR 2475), and RI detector (RI 2414). DMF (50 mM LiCl) was employed as eluent and molecular weights (M_n: number-average molecular weight) were calibrated relative to PEG (DMF). SEC Samples were prepared in DMF followed by filtration using GE Healthcare Whatman SPARTAN 13/0.2 RC 0.2 μm syringe filters. All reported values are relative to PEG standards, and are calculated from baseline to baseline to avoid artificially lowering the value of Đ and distorting the M_n relationships.

Ultraviolet-Visible Spectrometer. The absorbance of CTA in various solvents was measured on an S/N 5082 PerkinElmer Lambda 2 UV-Vis spectrometer.

Spectrometer. The wavelength range of the LEDs was measured by using an AvaSpec-2048 Fiber Optic Spectrometer, equipped with a 2048 pixel CCD Detector Array.

Photometer. The illuminance of the LEDs was measured with a PCE-LED 20 photometer equipped with a silicon photodiode sensor with filter, calibrated to a standard incandescent 2856 K lamp, and corrected to LED day white light spectrum. The intensity (mW/cm^2) was converted from the illuminance based on CIE luminous spectral efficiency (1983).

Experimental Section

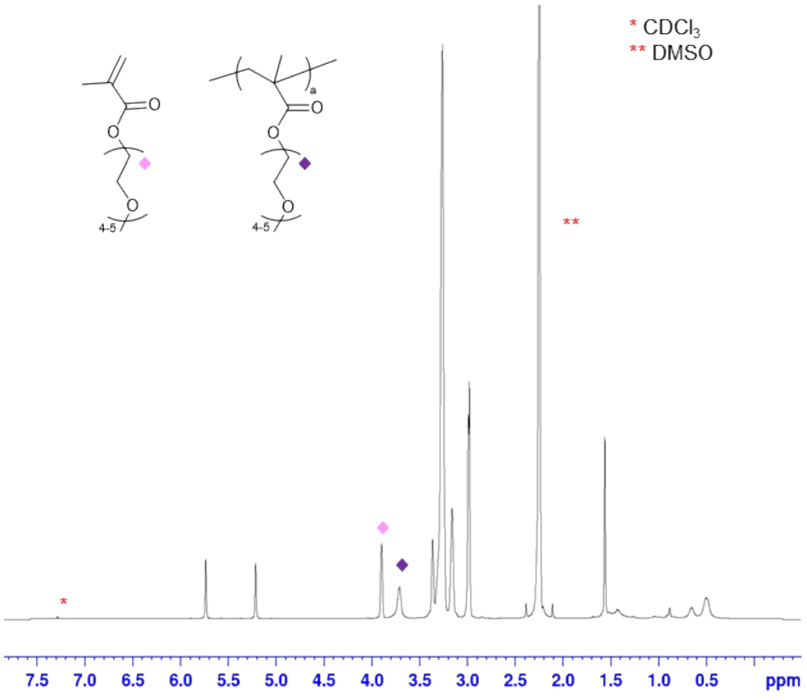

Figure S1. ^{1}H NMR spectrum of PEGMA monomer and P(PEGMA) in DMSO solution, measured in $CDCl_3$. The protons corresponding to monomer and polymer as annotated were used to calculate the conversion (**eq. 2**).

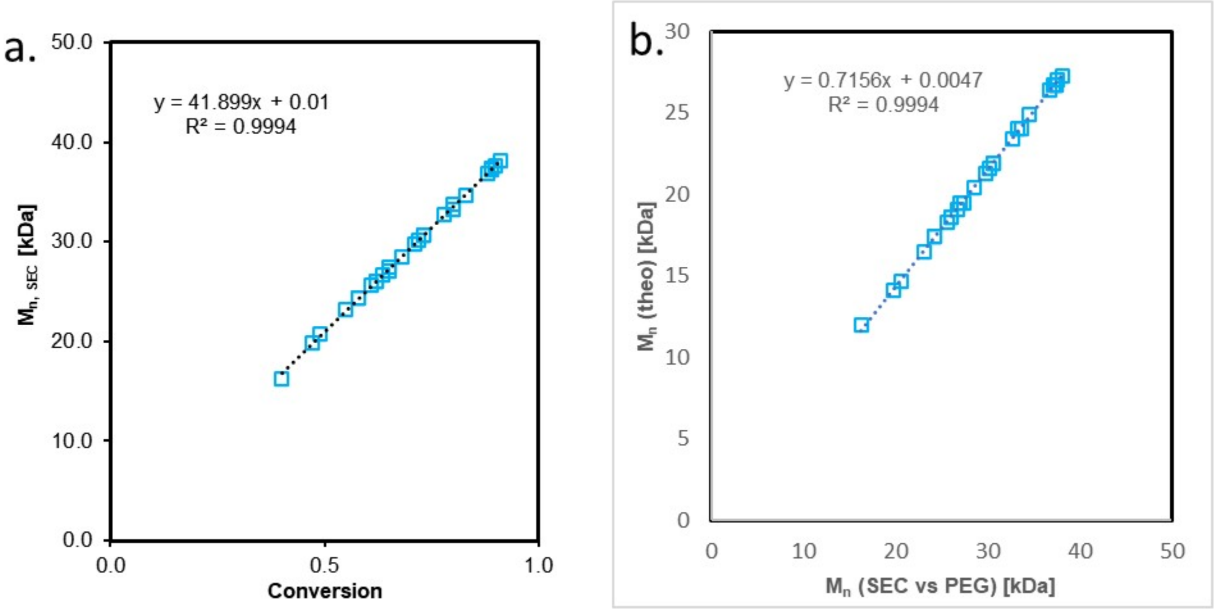

Figure S2. (a) Conversion plotted against $M_{n,\ SEC}$ calibrated against PEG standards for all SEC measurements in this manuscript. (b) Raw $M_{n,\ SEC}$ (vs PEG standards) results vs $M_{n,\ theoretical}$ plot. The $M_{n,\ SEC}$ reported in the manuscript is against PEG standards, but this plot indicates the deviation is due P(PEGMA) having a lower expansion coefficient than PEG.

Table S1. Solvent physical properties

Solvent	Viscosity (η), 25 ˚C	Dielectric constant	Dipole moment (D)	Molar volume [L/mol]	Refractive index (n_D)*	Boiling point [˚C]

	[mPa·s]					
1,4-Dioxane	1.20	2.25	0.45	0.086	1.42	101
Anisole	0.99	4.30	1.38	0.109	1.52	154
THF	0.46	7.58	1.75	0.081	1.40	66
EtOH	1.10	24.55	1.66	0.058	1.36	78
MeOH	0.54	32.70	2.87	0.040	1.33	65
DMF	0.80	36.71	3.86	0.077	1.43	153
DMSO	1.99	46.68	4.10	0.071	1.48	189

* Data taken from standard measurement at the sodium D line (589 nm)

Table S2. Solvatochromic scales and Hildebrand solubility parameters

	Kamlet−Abraham−Taft [1]			Catalan [2]				miscellaneous	
Solvent	α	β	π*	SP	SdP	SA	SB	$E_T(30)$ [3] [kcal/mol]	δ_H [4] [MPa$^{1/2}$]
1,4-Dioxane	0	0.37	0.50	0.737	0.312	0	0.444	36.0	20.7
Anisole	0	0.22	0.73	0.820	0.543	0.084	0.922	37.2	19.8
THF	0	0.55	0.58	0.714	0.634	0	0.591	37.4	18.6
EtOH	0.83	0.77	0.54	0.633	0.783	0.400	0.658	51.9	26.0
MeOH	0.93	0.62	0.60	0.608	0.904	0.605	0.545	55.4	29.7
DMF	0	0.69	0.88	0.759	0.977	0.031	0.613	43.2	24.1
DMSO	0	0.76	1.00	0.83	1.000	0.072	0.647	45.1	24.5

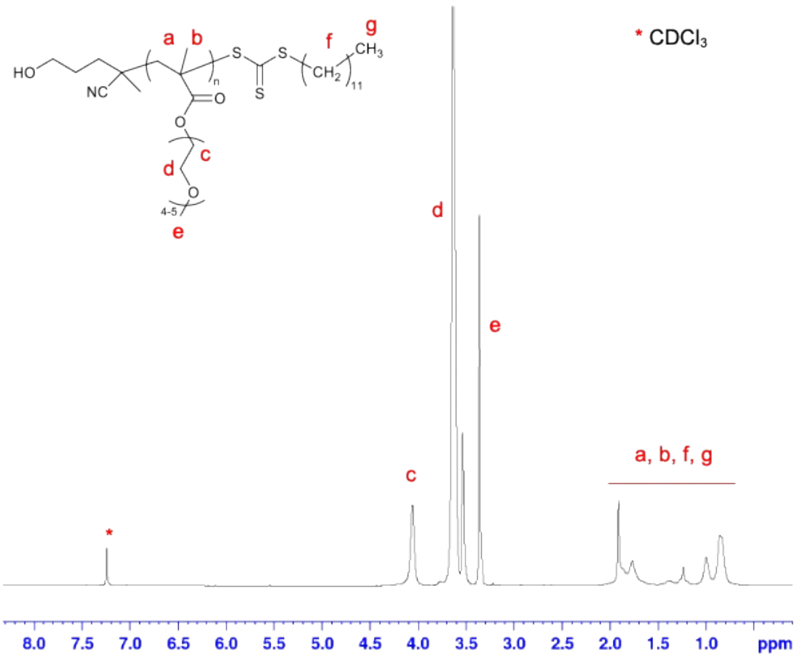

Figure S3. ^{1}H NMR of isolated P(PEGMA) in $CDCl_3$.

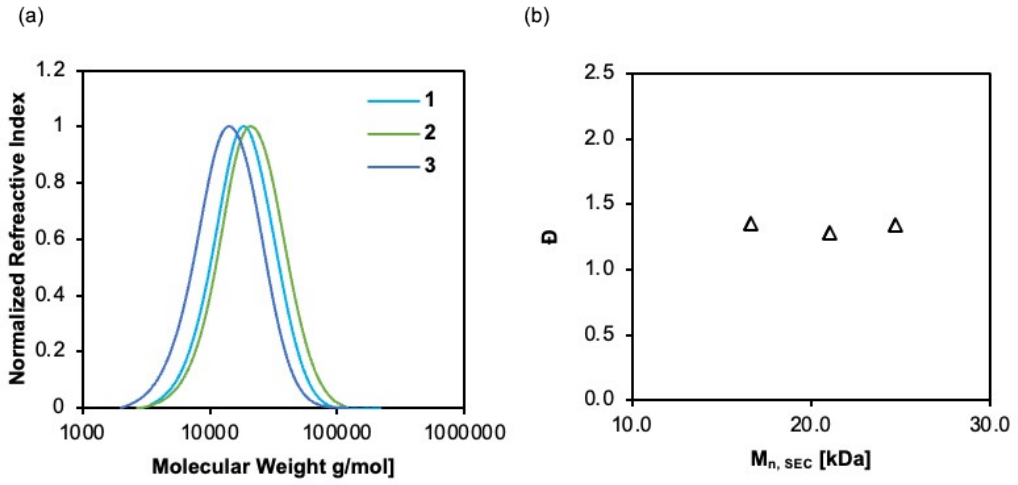

Figure S4. (a) Molecular weight distribution of P(PEGMA) synthesised under different wavelengths (1.6 mW/cm^2, 22 °C, 50 vol%). P(PEGMA) **1** irradiated under λ_{max} = 470 nm; **2** irradiated under λ_{max} = 515 nm; **3** irradiated under λ_{max} = 470 nm for 0.5 h, followed with subsequent irradiance under λ_{max} = 515 nm. (b) Đ vs $M_{n,\ SEC}$ plot. Eluent: DMF. Molecular weights were calibrated relative to PEO/PEG (DMF).

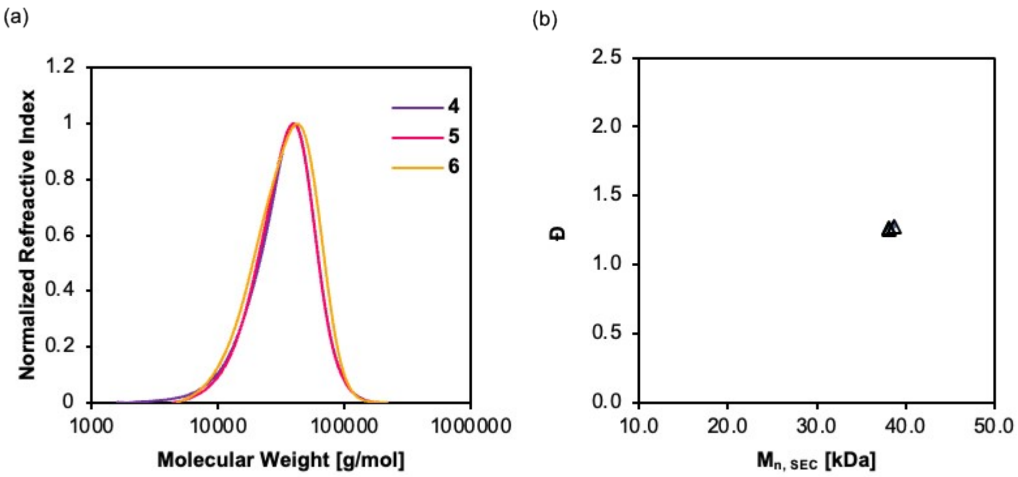

Figure S5. (a) Molecular weight distribution of P(PEGMA) synthesised at different monomer concentrations: 23 vol% (**4**), 17 vol% (**5**), and 9 vol% (**6**). Irradiation under λ_{max} = 470 nm, 1.6 mW/cm^2, 22 °C. (b) Đ vs $M_{n,\,SEC}$ plot. Eluent: DMF. Molecular weights were calibrated relative to PEO/PEG (DMF).

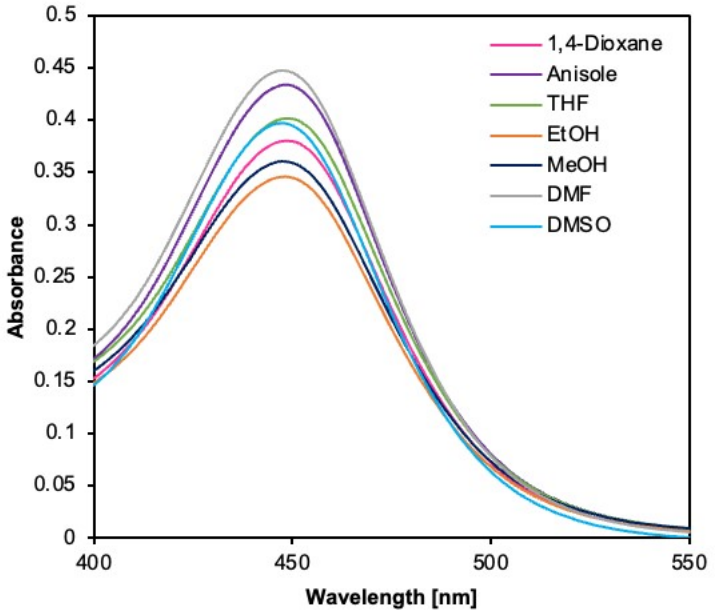

Figure S6. Absorbance of CTA corresponding to n→π* transition in different solvents (5 mg/mL).

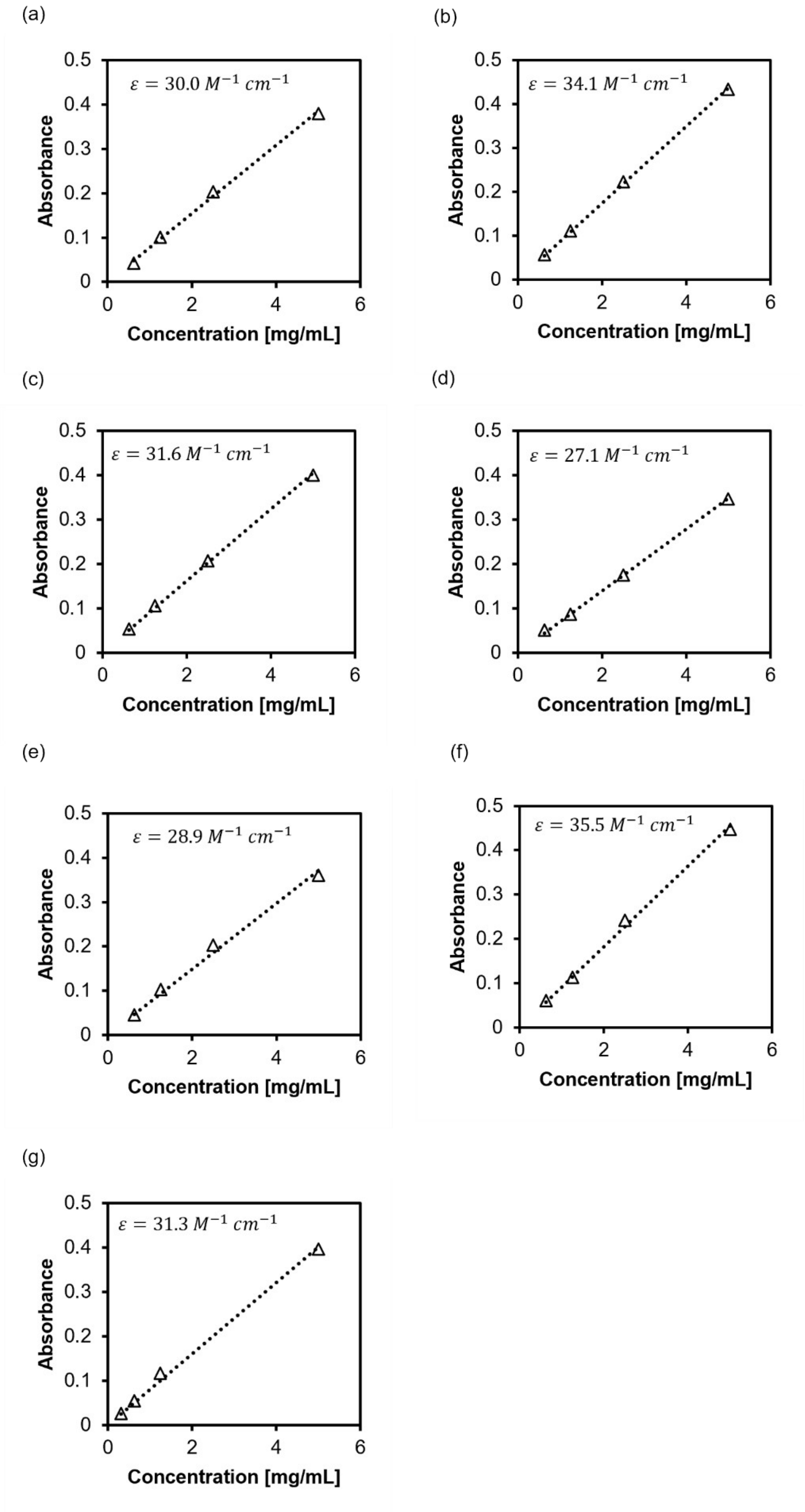

Figure S7. Beer-Lambert plots for the n→π* transition of CTA in (a) 1,4-dioxane, (b) anisole, (c) THF, (d) EtOH, (e) MeOH, (f) DMF, and (g) DMSO. Extinction coefficients were determined at the maximum absorbance at 447 nm.

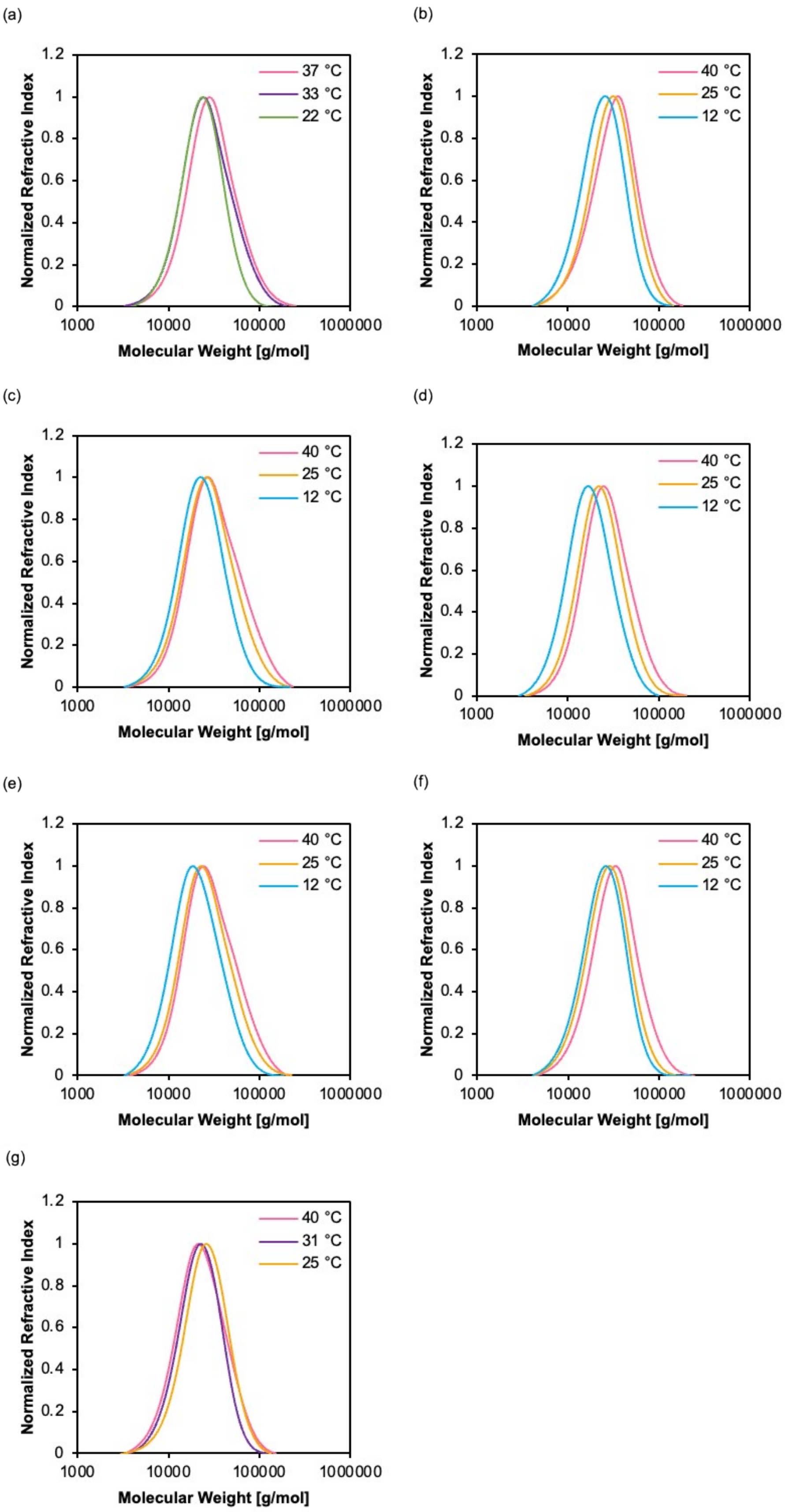

Figure S8. Molecular weight distribution of P(PEGMA) synthesised at different temperatures and in various solvents: (a) 1,4-dioxane, (b) anisole, (c) THF, (d) EtOH, (e) MeOH, (f) DMF, and (g) DMSO. Initiated under λ_{max} = 450 nm, 18 mW/cm^2 in DMSO, $[M]_0/[I]$=100, 50 vol%.

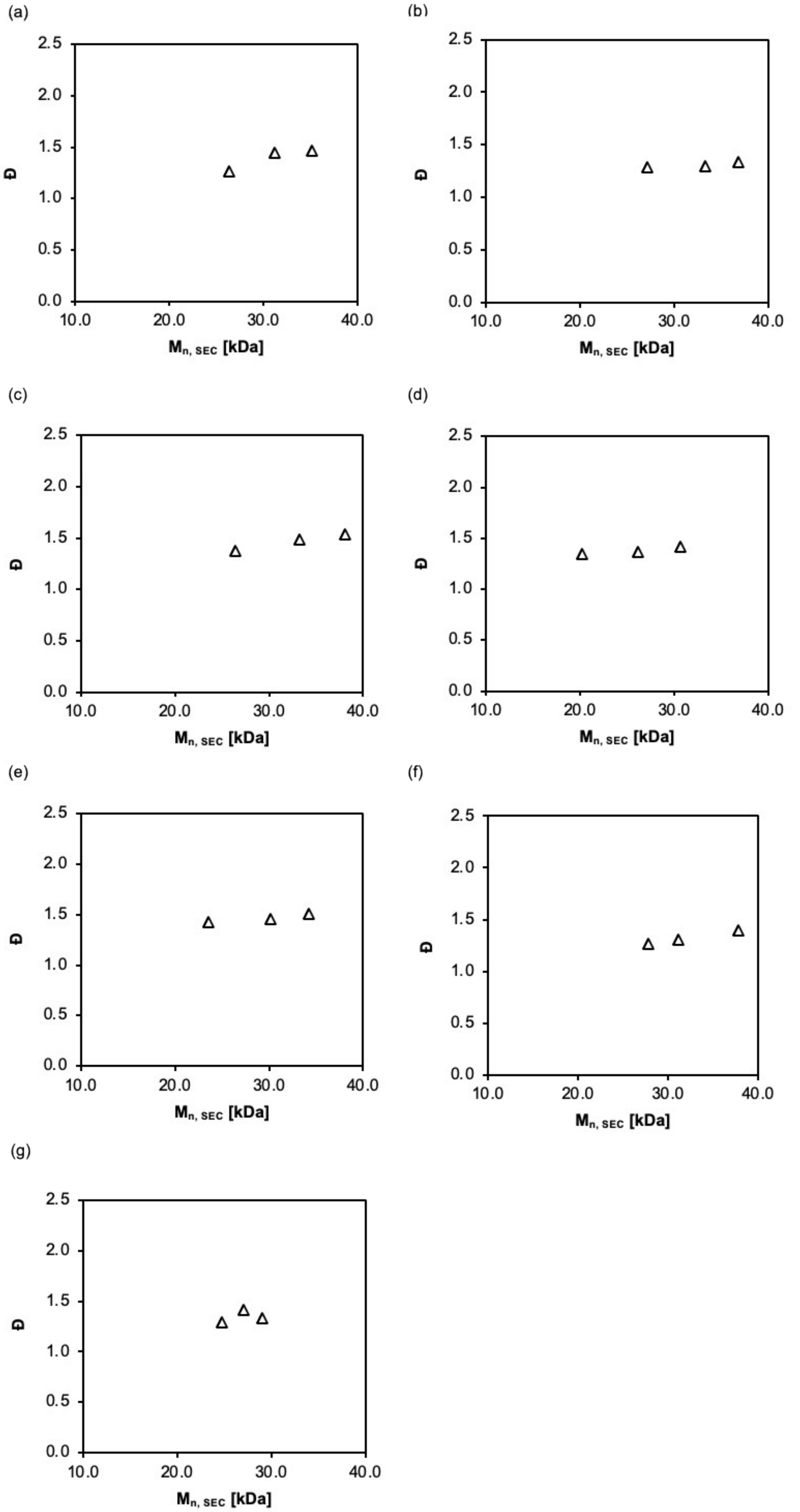

Figure S9. Đ vs $M_{n,\,SEC}$ plot. of P(PEGMA) synthesised at different temperatures and in various solvents: (a) 1,4-dioxane, (b) anisole, (c) THF, (d) EtOH, (e) MeOH, (f) DMF, and (g) DMSO. Initiated under λ_{max} = 450 nm, 18 mW/cm^2 in DMSO, $[M]_0/[I]$=100, 50 vol%.

Table S3. Adjusted R^2 values of simple linear regression.

	ε	Dielectric constant	Dipole moment (D)	Viscosity (η), 25 ˚C [mPa·s]	Molar volume [L/mol]	Refractive index (n_D)*	Boiling point [˚C]	$E_T(30)$ [kcal/mol]	δ_H [$MPa^{1/2}$]
k_p (25 ˚C)* [L/(mol·s)]	0.23 (p = 0.16)	-0.17 (p = 0.75)	-0.15 (p = 0.66)	0.16 (p = 0.20)	0.17 (p = 0.19)	0.39 (p = 0.08)	0.65 (p = 0.02)	-0.04 (p = 0.42)	-0.14 (p = 0.64)
k_p (40 ˚C) [L/(mol·s)]	0.36 (p = 0.09)	-0.20 (p = 0.91)	-0.17 (p = 0.71)	0.14 (p = 0.21)	0.37 (p = 0.08)	0.62 (p = 0.02)	0.73 (p = 0.01)	0.11 (p = 0.25)	-0.04 (p = 0.42)
E_a [kJ/mol]	0.43 (p =0.06)	-0.17 (p = 0.75)	0.06 (p = 0.29)	-0.19 (p = 0.88)	-0.06 (p = 0.45)	0.14 (p = 0.22)	0.07 (p = 0.29)	-0.10 (p = 0.53)	-0.13 (p = 0.60)
A [L/(mol·s)]	0.91 ** (p = 0.03)	-0.20 (p = 0.99)	-0.10 (p = 0.52)	-0.19 (p = 0.84)	-0.08 (p = 0.49)	0.01 (p = 0.35)	-0.05 (p = 0.43)	0.01 (p = 0.34)	-0.11 (p = 0.56)
ΔH‡ [kJ/mol]	0.43 (p = 0.06)	-0.17 (p = 0.75)	0.07 (p = 0.29)	-0.19 (p = 0.89)	-0.06 (p = 0.45)	0.14 (p = 0.22)	0.07 (p = 0.28)	-0.10 (p = 0.53)	-0.13 (p = 0.61)
ΔS‡ [J/(mol·K)]	0.56 (p = 0.03)	-0.17 (p = 0.72)	0.08 (p = 0.27)	-0.20 (p = 1.00)	0.00 (p = 0.36)	0.26 (p = 0.14)	0.20 (p = 0.18)	-0.07 (p = 0.47)	-0.12 (p = 0.57)

* k_p at 25 ˚C of 1,4-dioxane was calculated from the Arrhenius equation

** Pre-exponential factor A was fitted to an exponential model. Other parameters were analyzed with simple linear regression.

Table S4. Result of multivariate linear regression using the Kamlet-Abraham-Taft equation (**eq 11**). Error indicated as the standard error.

Property	x_0	a	b	s	Error DoF	Adjusted R^2	p-value	Notes
$\ln(k_p)$ (25 ˚C) [L/(mol·s)]	-3.84 (±0.63)	0.13 (±0.45)	0.38 (±0.86)	-1.24 (±1.02)	3	0.109	0.43	a, b, and s are insignificant
$\ln(k_p)$ (40 ˚C) [L/(mol·s)]	-3.42 (±0.55)	0.16 (±0.39)	0.66 (±0.74)	-1.41 (±0.88)	3	0.41	0.25	a, b, and s are insignificant
E_a [kJ/mol]	35.76 (± 11.23)	-0.77 (±7.96)	13.68 (±15.24)	-24.88 (±18.10)	3	0.08	0.45	a, b, and s are insignificant
A [L/(mol·s)]	1.17×10^4 (± 2.24×10^4)	3.80×10^3 (±1.59×10^4)	2.52×10^4 (± 3.05×10^4)	-3.15×10^4 (± 3.62×10^4)	3	-0.03	0.52	x_0, a, b, and s are insignificant
ΔH‡ [kJ/mol]	33.36 (± 11.25)	-0.78 (±7.97)	13.65 (±15.25)	-28.93 (±18.12)	3	0.08	0.45	x_0, a, b, and s are insignificant

ΔS‡ [J/(mol·K)]	-165.73 (± 33.24)	-1.66 (± 23.57)	44.49 (± 45.12)	-93.01 (± 53.60)	3	0.31	0.31	a, b, and s are insignificant

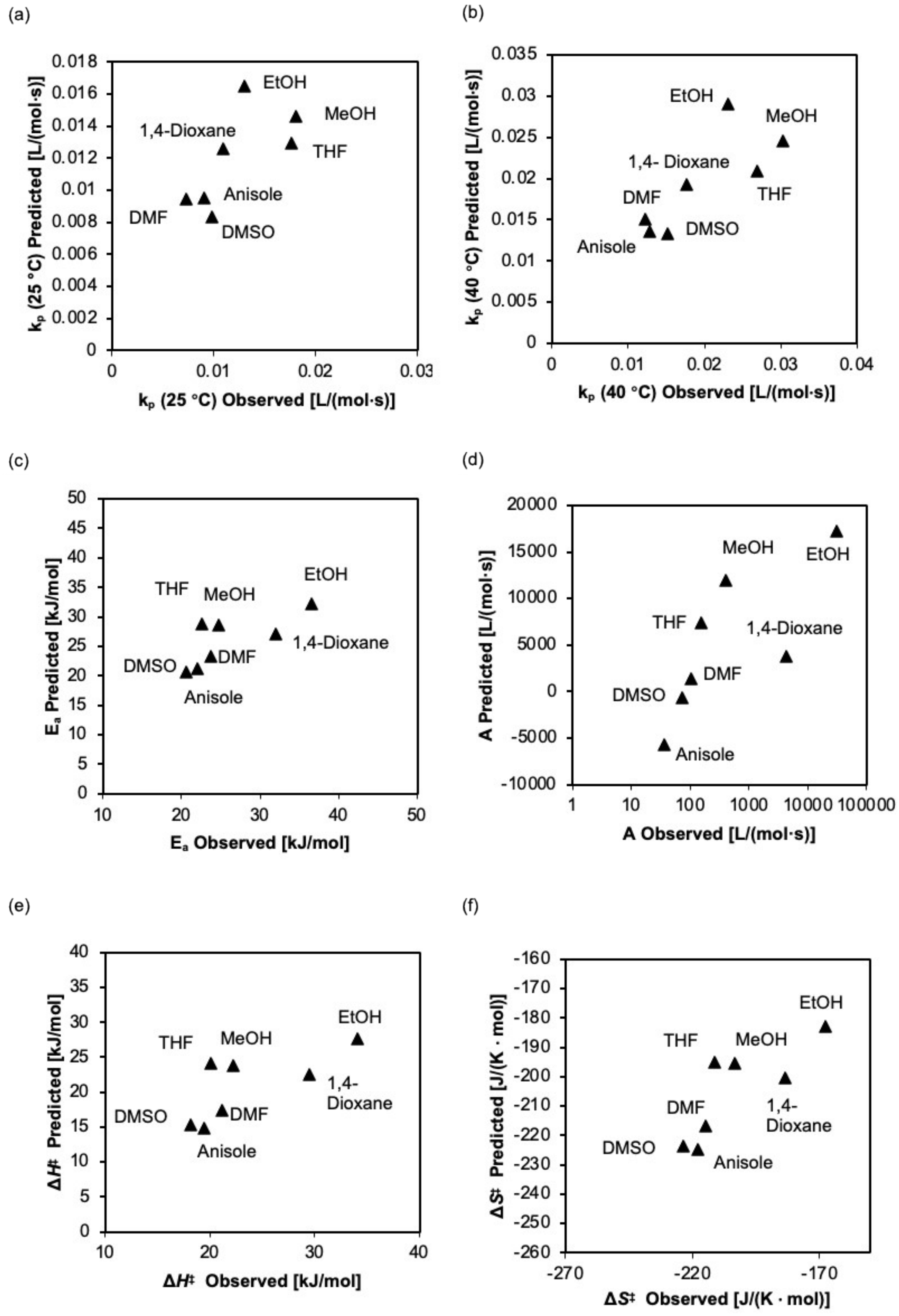

Figure S10. Predicted values of k_p, Arrhenius parameters, enthalpy and entropy of activation from multivariate linear regression using the Kamlet-Abraham-Taft equation (**eq 11**).

Table S5. Result of multivariate linear regression using the Catalan equation (**eq 12**). Error indicated as the standard error.

Property	x_0	p	d	a	b	Error DoF	Adjusted R^2	p-value	Notes
E_a [kJ/mol]	-2.82 (±62.91)	27.65 (± 74.21)	-30.93 (± 21.06)	22.07 (±26.37)	51.04 (± 38.362)	2	0.097	0.51	x_0, p, d, a, and b are insignificant
A [L/(mol·s)]	-7.97×10^4 (± 1.50×10^5)	8.26×10^4 (± 1.77×10^5)	-4.93×10^4 (± 5.02×10^4)	5.08×10^4 (± 6.28×10^4)	9.62×10^4 (± 9.14×10^4)	2	-0.43	0.73	x_0, p, d, a, and b are insignificant
$\Delta H^\ddagger$ [kJ/mol]	-4.85 (± 63.04)	27.16 (± 74.38)	-30.90 (± 21.11)	21.95 (± 26.42)	50.85 (± 38.45)	2	0.08	0.51	x_0, p, d, a, and b are insignificant
$\Delta S^\ddagger$ [J/(mol·K)]	-287.14 (± 173.28)	75.26 (±204.42)	-106.89 (± 58.01)	75.94 (± 72.62)	175.04 (±105.67)	2	0.418	0.35	x_0, p, d, a, and b are insignificant

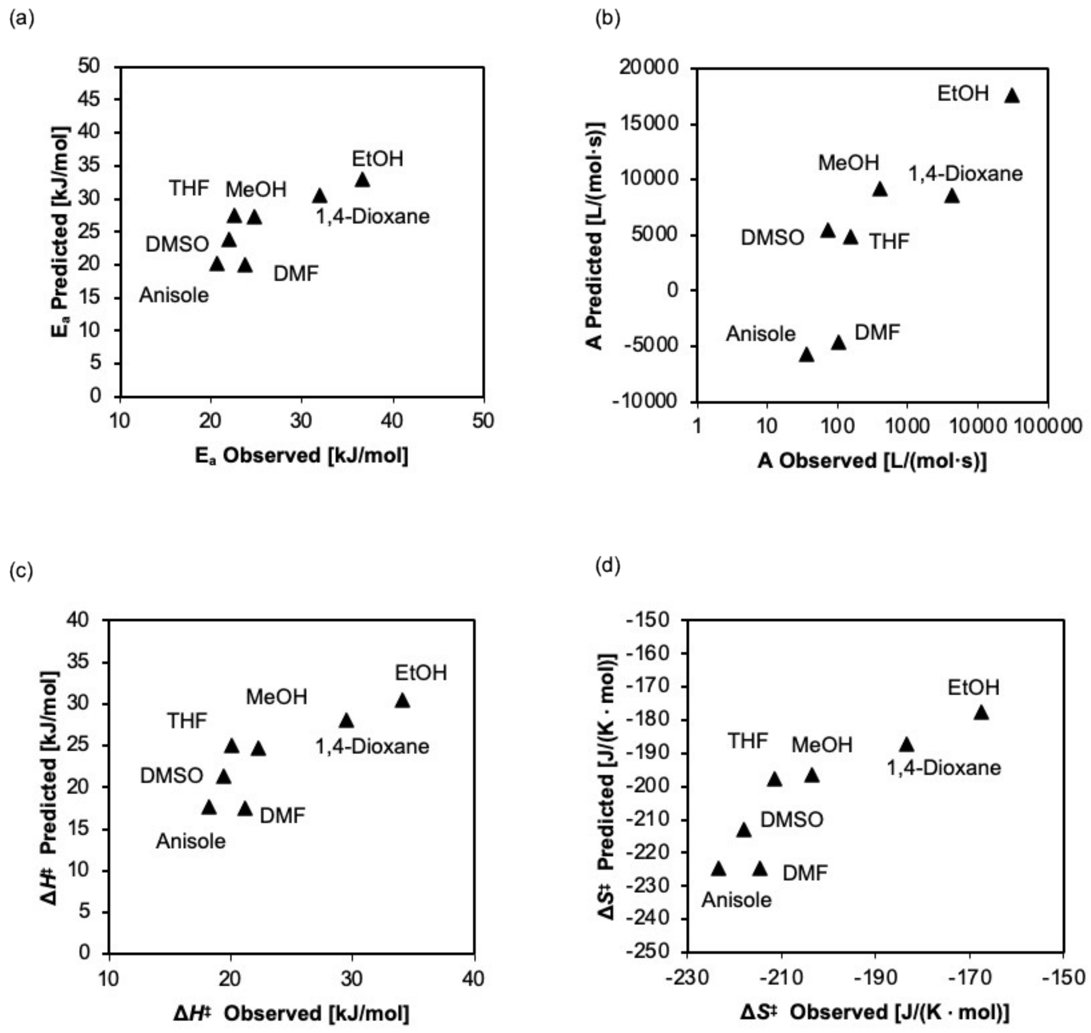

Figure S11. Predicted values of Arrhenius parameters, enthalpy and entropy of activation from multivariate linear regression using the Catalan equation (**eq 12**).

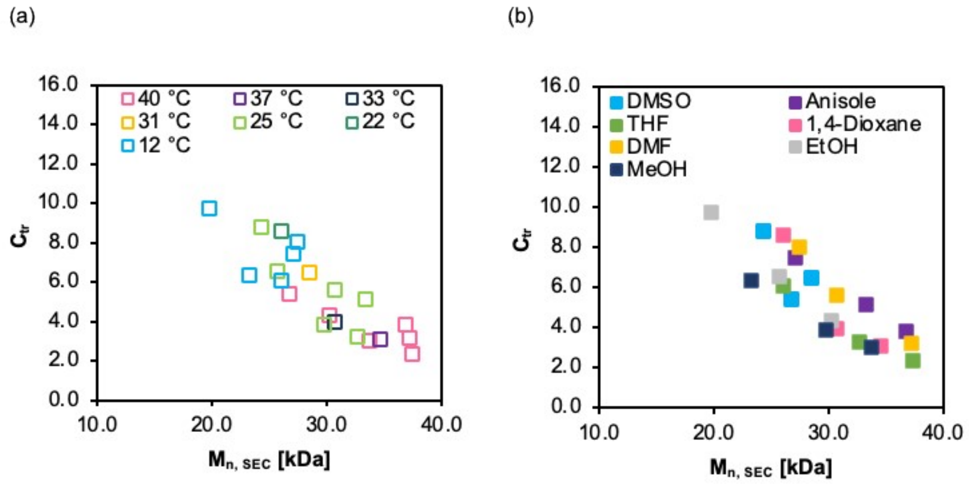

Figure S12. C_{tr} vs $M_{n,\,SEC}$ for P(PEGMA) **7-27** synthesised at various temperatures (a) and in various solvents (b).

Reference

1. M. J. Kamlet, J. L. M. Abboud, M. H. Abraham and R. W. Taft, *The Journal of Organic Chemistry*, 1983, **48**, 2877-2887.
2. J. Catalán, *Journal of Physical Chemistry B*, 2009, **113**, 5951-5960.
3. K. Dimroth, C. Reichardt, T. Siepmann and F. Bohlmann, *Justus Liebigs Annalen der Chemie*, 1963, **661**, 1-37.
4. A. F. M. Barton, ed., *CRC Handbook of Solubility Parameters and Other Cohesion Parameters : Second Edition*, Taylor and Francis, London, 2017.
